# *Listeria monocytogenes* Relies on the Heme-Regulated Transporter *hrtAB* to Resist Heme Toxicity and Uses Heme as a Signal to Induce Transcription of *lmo1634*, Encoding *Listeria* Adhesion Protein

**DOI:** 10.3389/fmicb.2018.03090

**Published:** 2018-12-18

**Authors:** Patrícia Teixeira dos Santos, Pernille Tholund Larsen, Pilar Menendez-Gil, Eva Maria Sternkopf Lillebæk, Birgitte Haahr Kallipolitis

**Affiliations:** Department of Biochemistry and Molecular Biology, University of Southern Denmark, Odense, Denmark

**Keywords:** *Listeria monocytogenes*, heme toxicity, heme detoxification, *hrtAB*, LAP

## Abstract

For pathogenic bacteria, host-derived heme represents an important metabolic cofactor and a source for iron. However, high levels of heme are toxic to bacteria. We have previously shown that excess heme has a growth-inhibitory effect on the Gram-positive foodborne pathogen *Listeria monocytogenes*, and we have learned that the LhrC1-5 family of small RNAs, together with the two-component system (TCS) LisRK, play a role in the adaptation of *L. monocytogenes* to heme stress conditions. However, a broader knowledge on how this pathogen responds to heme toxicity is still lacking. Here, we analyzed the global transcriptomic response of *L. monocytogenes* to heme stress. We found that the response of *L. monocytogenes* to excess heme is multifaceted, involving various strategies acting to minimize the toxic effects of heme. For example, heme exposure triggers the SOS response that deals with DNA damage. In parallel, *L. monocytogenes* shuts down the transcription of genes involved in heme/iron uptake and utilization. Furthermore, heme stress resulted in a massive increase in the transcription of a putative heme detoxification system, *hrtAB*, which is highly conserved in Gram-positive bacteria. As expected, we found that the TCS HssRS is required for heme-mediated induction of *hrtAB* and that a functional heme efflux system is essential for *L. monocytogenes* to resist heme toxicity. Curiously, the most highly up-regulated gene upon heme stress was *lmo1634*, encoding the *Listeria* adhesion protein, LAP, which acts to promote the translocation of *L. monocytogenes* across the intestinal barrier. Additionally, LAP is predicted to act as a bifunctional acetaldehyde-CoA/alcohol dehydrogenase. Surprisingly, a mutant lacking *lmo1634* grows well under heme stress conditions, showing that LAP is not required for *L. monocytogenes* to resist heme toxicity. Likewise, a functional ResDE TCS, which contributes to heme-mediated expression of *lmo1634*, is not required for the adaptation of *L. monocytogenes* to heme stress conditions. Collectively, this study provides novel insights into the strategies employed by *L. monocytogenes* to resist heme toxicity. Our findings indicate that *L. monocytogenes* is using heme as a host-derived signaling molecule to control the expression of its virulence genes, as exemplified by *lmo1634*.

## Introduction

*Listeria monocytogenes* is a Gram-positive, facultative anaerobe, closely related to other bacterial species such as *Bacillus*, *Clostridium, Enterococcus, Streptococcus*, and *Staphylococcus* (Vazquez-Boland et al., 2001). This opportunistic foodborne pathogen is the causative agent of listeriosis, which can turn into a life-threatening disease for immunocompromised patients, elderly, neonates and pregnant women (Vazquez-Boland et al., 2001; Swaminathan and Gerner-Smidt, 2007; Cossart and Lebreton, 2014). Upon ingestion of contaminated food, *L. monocytogenes* may cross the intestinal barrier and reach the lymph and bloodstream, allowing its dissemination throughout the host organism, which will eventually lead to bacteremia and septicemia (Dussurget, 2008; Allerberger and Wagner, 2010; Cossart and Lebreton, 2014). When in the bloodstream, *L. monocytogenes* has the ability to secrete listeriolysin O (LLO), which will lyse the erythrocytes and enable the release of hemoglobin (Parrisius et al., 1986; Geoffroy et al., 1987; Foller et al., 2007), a situation that can lead to levels of free heme as high as 20 μM (Arruda et al., 2004). Due to the reactive nature of heme, invading pathogens must protect themselves against the potential damaging effects of this molecule in heme-rich environments (McLaughlin et al., 2011; Choby and Skaar, 2016; Huang and Wilks, 2017). For *L. monocytogenes*, the mechanism by which this pathogen senses and responds to excess heme is far from being understood. Our most recent study showed that excess heme affects the growth of *L. monocytogenes*, and that the LhrC family of small RNAs, as well as the two-component system (TCS) response regulator LisR, contribute to the adaptation of *L. monocytogenes* to excess heme (Dos Santos et al., 2018). However, no other major contributors to the prevention of heme toxicity have been identified so far in *L. monocytogenes*. Interestingly, in the closely related pathogen *Staphylococcus aureus*, a heme detoxification system has been widely described (Stauff et al., 2007; Torres et al., 2007; Wakeman et al., 2014). The TCS HssRS (Heme-Sensor System) responds to heme exposure and activates the expression of the Heme Regulated Transporter, HrtAB, which protects the bacteria from heme toxicity by exporting heme (Torres et al., 2007; Stauff and Skaar, 2009b). Orthologs of this system have been identified and studied in other Gram-positive bacteria including *Bacillus anthracis* (Stauff and Skaar, 2009a), *B. thuringiensis* (Schmidt et al., 2016), *Lactococcus lactis* (Pedersen et al., 2008; Lechardeur et al., 2012), *Streptococcus agalactiae* (Fernandez et al., 2010; Joubert et al., 2017), *Corynebacterium diphtheria* and Group A *Streptococcus* (Sachla et al., 2014), suggesting that specific mechanisms for resisting heme stress are a common requirement for these bacteria. The HssRS system and HrtAB exporter are conserved in *L. monocytogenes* as well (Torres et al., 2007); however, their roles in dealing with heme toxicity remain to be clarified.

In the present study, we made use of RNA-sequencing (RNA-seq) to explore the global transcriptomic response of *L. monocytogenes* to heme stress. Our analysis revealed that *L. monocytogenes* responds to heme stress by inducing various stress responses known to deal with oxidative stress and DNA damage, including the SOS response. Also, *L. monocytogenes* reacts to excess heme by shutting down the expression of genes involved in heme/iron uptake and utilization. Our study revealed the importance of the predicted players in preventing heme toxicity: the TCS HssRS and the efflux pump HrtAB. Indeed, the *hrtA* and *hrtB* genes correspond to the 2nd and 3rd most up-regulated genes in *L. monocytogenes* in response to heme exposure. In line with this, we demonstrated that a functional heme detoxification system is essential for the adaptation of *L. monocytogenes* to heme stress conditions. Curiously, the most highly up-regulated gene in response to heme exposure was *lmo1634*, encoding LAP, the *Listeria* adhesion protein. To our surprise, we found that *lmo1634* is dispensable for growth *of L. monocytogenes* under heme stress conditions. These findings made us speculate that *L. monocytogenes* uses heme as a host-derived signaling molecule to modulate the transcription of *lmo1634* and, possibly, other heme-regulated virulence genes in blood-rich environments.

## Materials and Methods

### Bacterial Strains and Growth Conditions

The wild-type strains used in this study were *L. monocytogenes* serotype 1/2a strain EGD-e (ATCC BAA-679), *L. monocytogenes* serotype 1/2c strain LO28 (Vazquez-Boland et al., 1992), and *L. monocytogenes* serotype 1/2a strain EGD (Larsen et al., 2006). The mutant derivatives carrying in-frame deletions of *resD* (EGDΔ*resD*) and *lisR* (LO28Δ*lisR*) were constructed in previous work in the EGD or LO28 wild-type strains (Kallipolitis et al., 2003; Larsen et al., 2006). The remaining in-frame deletion mutant derivatives (EGD-eΔ*lmo1634*, LO28Δ*hssR* and LO28Δ*hrtA*) were constructed in EGD-e or LO28 in the present study by using the temperature-sensitive shuttle vector pAUL-A (Schaferkordt and Chakraborty, 1995) as described previously (Christiansen et al., 2004). Primers used for constructing the in-frame deletions of *lmo1634*, *hssR* and *hrtA* are listed in Supplementary Table S1. *L. monocytogenes* was routinely grown in brain heart infusion broth (BHI, Oxoid) at 37 °C with aeration. When appropriate, cultures were supplemented with kanamycin (50 μg/mL) or erythromycin (5 μg/mL). For heme-induced transcription, cultures were exposed to hemin (Sigma), which is the commercially available version of heme. Hemin contains the oxidized Fe^3+^ ferric form instead of the reduced Fe^2+^ ferrous form present in heme. Throughout the manuscript, we refer to hemin for experimental purposes, while for general discussions we will refer to heme. Hemin was dissolved in 1.4 M NaOH and for each experiment, stock solutions were freshly prepared. In hemin stress assays, overnight cultures were diluted to OD_600_ = 0.002 into BHI adjusted with various concentrations of hemin. Alternatively, overnight cultures were diluted to OD_600_ = 0.002 into BHI and hemin was added to exponentially growing cells (OD_600_ = 0.2); growth was monitored until strains reached stationary phase, or single OD_600_ measurements were taken at the indicated time points. Each assay was performed in biological triplicates. For cloning of plasmid vectors, *Escherichia coli* TOP10 cells (Invitrogen) were grown with aeration in Luria-Bertani (LB) broth. When required, the LB broth was supplemented with kanamycin (50 μg/mL) or erythromycin (150 μg/mL).

### Plasmid Constructions and β-Galactosidase Assays

To study the transcriptional activity of *hrtAB*, we used the promoter-less *lacZ* transcriptional fusion vector pTCV-lac (Poyart and Trieu-Cuot, 1997) fused to the wild-type or the mutated promoter region of the *hrtAB*-encoding gene, obtained by PCR from LO28 chromosomal DNA. All primers used for constructing the transcriptional fusions are listed in Supplementary Table S1. The pTCV-*lmo2210*-*lacZ* plasmid was constructed in previous work (Gottschalk et al., 2008). For the β-galactosidase assay, overnight cultures of *L. monocytogenes* LO28 strains carrying the plasmids were diluted to OD_600_ = 0.02 in fresh BHI and grown to OD_600_ = 0.35. Then, the cultures were split and stressed with 8 μM hemin for 1 or 2 h. Samples (1 mL) were withdrawn prior to hemin stress and at various time points after the onset of hemin exposure. β-galactosidase assays were conducted as described in a previous study (Christiansen et al., 2004). As heme exposure resulted in impaired growth relative to the non-exposed control condition, a direct comparison between the heme-stressed condition and non-stressed control condition was not possible. However, the growth of the wild-type and mutant strains was comparable under each of the conditions tested (i.e., control or heme exposure, respectively). Consequently, the β-galactosidase activities of *L. monocytogenes* LO28 wild-type and mutant strains were analyzed for each of the conditions using two-tailed Student’s *t*-test (i.e., wild-type, hemin stressed vs. mutant, hemin stressed). Differences with at least 95% confidence were considered statistically significant.

### Total RNA Isolation and Purification

For primer extension and northern blot analysis, *L. monocytogenes* strains were grown to OD_600_ = 0.35–0.4. Then, the cultures were split, and cells were exposed to the indicated concentrations of hemin. At the indicated time points, 10 mL samples were drawn from the cultures. To harvest the cells, the samples were centrifuged at 11,000 rcf for 3 min at 4°C, and the cell pellets were snap-frozen in liquid nitrogen. Then, cells were disrupted by the FastPrep instrument (Bio101, Thermo Scientific Corporation) and total RNA was extracted using TRI Reagent (Molecular Research Center, Inc.) as described in a previous study (Nielsen et al., 2010). The integrity, concentration and purity of the RNA were confirmed by agarose gel electrophoresis and DeNovix DS-11 Fx+.

For transcriptomic analysis, *L. monocytogenes* EGD-e wild-type strain was grown to OD_600_ = 0.4. Cultures were then split, half stressed with 8 μM hemin, and samples were taken (5 mL) after 30 min (done in biological triplicates). RNAprotect^®^ Cell Reagent (Qiagen) was added (10 mL), the cultures homogenized and incubated 5 min at room temperature. Cells were harvested by centrifugation at 5,500 rcf for 5 min at 4°C, and snap-frozen in liquid nitrogen. The pellets were re-suspended in 1 mL of TRIzol^TM^ Reagent (Ambion) and cells disrupted by the FastPrep instrument (Bio101, Thermo Scientific Corporation). Total RNA was subsequently isolated as recommended by the manufacturer. The integrity, concentration and purity of the RNA were confirmed by agarose gel electrophoresis and DeNovix DS-11 Fx+.

### RNA Integrity, rRNA Depletion and Library Preparation

RNA integrity and quantification, ribosomal RNA depletion, library preparation and sequencing were performed by GenXPro GmbH (Frankfurt, Germany). The quality of RNA was assessed using Labchip GX II bioanalyzer (Perkin Elmer). To remove DNA contamination, total RNA was incubated with Baseline-Zero DNase (Epicenter) in the presence of RiboLock RNase inhibitor (40 U/μl) (Thermo Fisher Scientific) for 30 min at 37°C followed by purification using Zymo-Spin column (Zymo Research). Briefly, the samples were mixed with 2 volumes of RNA Binding Buffer and added to an equal volume of ≥99.8% ethanol (Roth). The mixture was vortexed and transferred to Zymo-Spin^TM^ IC Column and centrifuged at 12,000 rcf. The RNA bound to the column was washed twice with RNA Wash Buffer then RNA was eluted in RNase free water. Concentration of RNA was measured using fluorescence-based Qubit^TM^ RNA HS Assay (Thermo Fisher Scientific). To enrich mRNA and remove ribosomal RNA (rRNA) from total RNA, total RNA was treated with Ribo-Zero rRNA removal kit (Illumina). Briefly, beads were washed twice and hybridized with probes at 68°C for 10 min. A total of 500 ng RNA was added to the mixture and incubated at room temperature and 50°C for 5 min each, followed by separation of mRNA from rRNA, which was bound to the beads using magnetic stand. Enriched mRNA was purified by Zymo-Spin column (Zymo Research) and run on Labchip GX II bioanalyzer (Perkin Elmer) to confirm reduction of rRNA. Preparation of cDNA fragment libraries was performed using the NEBNext^®^ Ultra^TM^ II Directional RNA Library Prep Kit for Illumina^®^ (Illumina) with minor modifications. Briefly, the enriched mRNA was fragmented for 15 min at 94°C and reverse transcribed to synthesize the first-strand cDNA followed by second strand cDNA synthesis. Double-stranded cDNA (ds cDNA) was purified using NucleoMag (Macherey nagel) SPRI selection. End repair was performed on the ds cDNA library followed by ligation of adaptors. After purification using NucleoMag SPRI beads, test qRT-PCR (Applied Biosystems) was performed using KAPA Hifi polymerase (Roche) with EvaGreen^®^ (Biotium) to determine appropriate cycle numbers for PCR. Using NEBNext Multiplex Oligos for Illumina (Dual Index Primers), high fidelity PCR was performed using KAPA Hifi polymerase to selectively enrich library fragments. The PCR products were purified twice using NucleoMag (Macherey nagel) SPRI beads and the quality of the final library was assessed on Labchip GX II bioanalyzer (Perkin Elmer).

### RNA Sequencing and Data Analysis

Indexed and purified libraries were loaded together onto a flow cell, and sequencing was carried out on the Illumina NextSeq 500 platform (paired-end, 2 × 75 bp per read). Sequenced reads were quality-checked using FastQC, and Illumina adapter sequences and low-quality base pairs were removed using cutadapt version 1.9 (Martin, 2011). Reads were mapped against the complete sequenced genome of the *L. monocytogenes* reference strain EGD-e (ENSEMBL ASM19603v1), using Bowtie 2 v 2.2.4 with standard parameters and sensitive-local (Langmead and Salzberg, 2012). BAM alignment files were used as input for read counting using htseq-count version 0.6.0. Differential expression (DE) analyses were performed using DESeq2 in R v 3.2.2 (Love et al., 2014), and the DE was reported as log_2_ fold changes. *p*-values were adjusted by the DESeq2 default Benjamini–Hochberg (BH) adjustment method. The fold changes were calculated by comparing expression levels of hemin-stressed vs. non-stressed. Genes with at least a twofold (>1 log_2_) change in expression and an adjusted *p*-value < 0.05 were considered as DE in the interpretation applied in this study. The transcriptomic data has been deposited on the Sequence Read Archive (SRA) database and is accessible through the SRA accession PRJNA491646.

### Primer Extension Analysis

Primer extension analysis was performed as described in a previous study from our laboratory (Christiansen et al., 2004). The ^32^P-labeled, single-stranded primers used for mapping the transcription start site of *hrtAB* are listed in Supplementary Table S1.

### Northern Blotting Analysis

For northern blotting analysis, total RNA (20 μg) was separated on a formaldehyde agarose gel for 3 h and 15 min prior to capillarity blotting on a Zeta-Probe membrane (Bio-Rad) (Sheehan et al., 1995). Membranes were hybridized with ^32^P-labeled DNA single-stranded probes; the probes used for northern blot analysis are listed in Supplementary Table S1. RNA bands were visualized using a Typhoon FLA9000 (GE Healthcare) and analyzed with IQTL 8.0 quantification software (GE Healthcare).

## Results

### The Transcriptome of *L. monocytogenes* Is Greatly Affected Upon Exposure to Heme Stress

To investigate how excess heme affects global gene expression in the widely studied *L. monocytogenes* strain EGD-e, RNA-seq was performed after exposing exponentially growing cells to a sub-inhibitory concentration of hemin (8 μM) for 30 min; hemin is the commercially available version of heme and its oxidized version. Non-stressed cells were included as a control and the transcriptomes of heme-stressed and non-stressed cells were compared. The RNA-seq analysis revealed that heme exposure caused 1101 genes to be differentially expressed (453 induced and 648 repressed) when compared to the unstressed condition, using a threshold of twofold and *p* < 0.05, corresponding to approximately 38% of all genes in the genome of *L. monocytogenes* (Supplementary Table S2). The RNA-seq data of 12 genes (including up-, non- and down-regulated genes) was validated by northern blot analysis (Supplementary Figure S1A). The relation between the RNA-seq data and the validation (biological triplicates) is shown in Supplementary Figures S1B–D.

**FIGURE 1:**
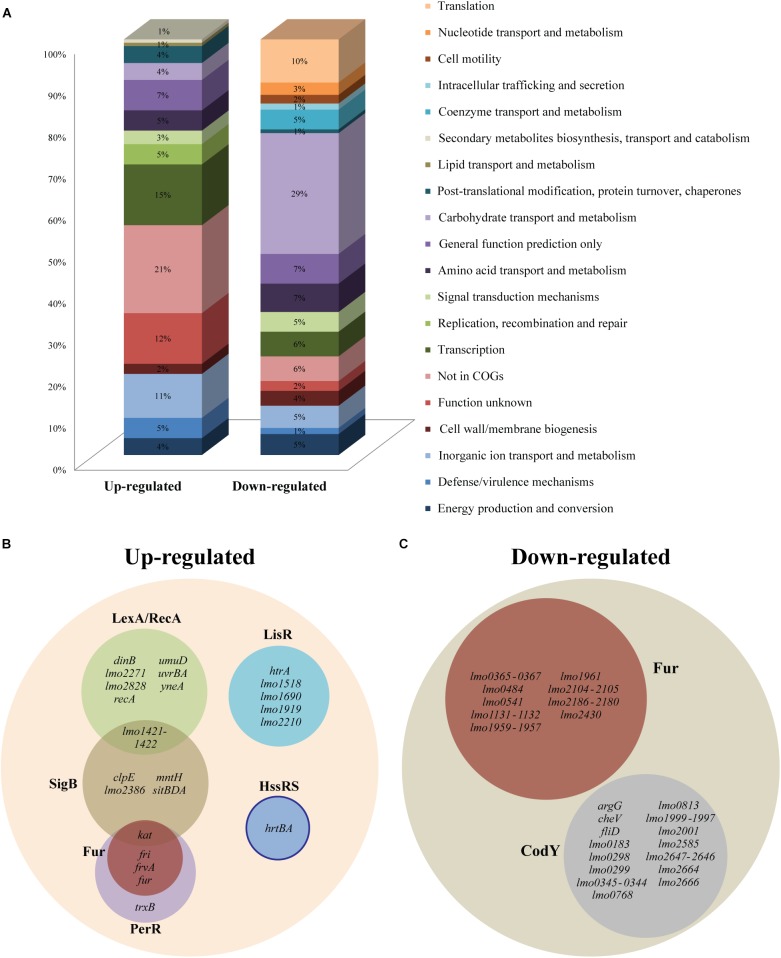
RNA-seq analysis of *Listeria monocytogenes* EGD-e exposed to heme stress. **(A)** Distribution of differentially expressed up- and down-regulated genes with at least 4.0-fold change (*p* < 0.05) according to the COG designation. **(B)** Up- and **(C)** down-regulated genes (at least 4.0-fold) grouped into different regulons. The different colors represent a different regulon and the overlaps of the different regulons are also shown. The distribution of genes into regulons was based on the studies referred to in Supplementary Table S3. The solid line represents a predicted regulon.

To further analyze the most prominent effects of heme stress, we focused on the genes that were at least fourfold up- or down-regulated by heme, corresponding to 110 and 297 genes, respectively (Supplementary Table S3). Going through the highly up-regulated genes, it was noticeable that a clear majority codes for hypothetical proteins (21%) or proteins with unknown function (12%), indicating that proteins with important roles in the adaptation of this pathogen to heme-rich host environments are still to be characterized (Figure 1A). A high proportion of transcriptional regulators (15%) also prevailed among the up-regulated genes, suggesting that a fast activation or repression of certain genes is required for the bacterium to adapt to conditions of excess heme. Regarding the highly down-regulated genes, 29% encode for proteins involved in carbohydrate transport and metabolism, and a lower but still significant number of genes (10%) code for proteins with roles in translation (Figure 1A).

### Heme Stress Remodels the Expression of Metabolic Genes, Indicating a Shift From Aerobic to Anaerobic Metabolism

Further analysis of the up- and down-regulated genes revealed an extensive overlap with central metabolic genes differentially transcribed during anaerobic growth (Muller-Herbst et al., 2014). In their study, Muller-Herbst et al. (2014) showed that 111 genes were down-regulated, and 28 genes were up-regulated in response to a shift from aerobic to anaerobic growth. Of the 111 genes, 85 were found to be down-regulated at least 2-fold upon hemin exposure, corresponding to 76% of the genes down-regulated anaerobically (Supplementary Tables S2, S3). Regarding the 28 up-regulated genes during anaerobic growth, 15 genes were at least twofold up-regulated in our study, including the most highly induced gene, *lmo1634* (434-fold) which is further analyzed below (Supplementary Tables S2, S3). Similarly, we observed an overlap between genes that were differentially expressed under low oxygen conditions (Toledo-Arana et al., 2009), and genes shown here to be highly up- or down-regulated in response to heme stress (Supplementary Table S3). Collectively, these findings suggest that the response of *L. monocytogenes* to heme toxicity involves a readjustment of its metabolic machinery, corresponding to a shift from aerobic to anaerobic metabolism.

### Heme Exposure Triggers a Variety of Stress Responses in *L. monocytogenes*, Including the SOS Response

Of the 110 up-regulated genes with a 4.0-fold cut-off, 38 could be allocated into known regulons, some of them under the control of more than one regulator (Figure 1B and Supplementary Table S3). The most represented regulon was LexA/RecA, which is known to control genes involved in the SOS response that copes with various types of DNA damage (van der Veen et al., 2010). Ten genes belonging to the LexA/RecA regulon were up-regulated more than 4.0-fold upon heme exposure (Figure 1B) and the entire regulon could be found among the genes that were more than 2.0-fold up-regulated (Supplementary Table S2). Among the most highly induced genes was the RecA-encoding gene (*lmo1398*) and genes encoding the cell division inhibitor YneA (*lmo1303*), the excinuclease UvrBA (*lmo2489*-*lmo2488*), and the translesion DNA polymerases DinB (*lmo1975*) and UmuD (*lmo2675*) (van der Veen et al., 2010). These findings clearly demonstrate that heme stress triggers the expression of SOS response genes known to promote the repair and survival of DNA-damaged cells. In addition to the LexA/RecA regulon, nine genes belonging to the SigmaB (SigB) regulon were induced at least fourfold in response to heme stress (Figure 1B). The alternative sigma factor SigB positively regulates genes involved in the response of *L. monocytogenes* to stress conditions such as heat, osmotic and acid stresses (Hain et al., 2008), and SigB has also been implicated in the oxidative stress response (Liu et al., 2017). Among the heme-induced genes belonging to the SigB regulon we found a bile-resistance system (*lmo1421-lmo1422*), which is regulated by LexA/RecA as well (Figure 1B). Interestingly, four genes known to be controlled by the ferric uptake regulator, Fur, and the peroxide resistance regulator, PerR, were greatly up-regulated in response to heme stress (Figure 1B), although most genes belonging to the Fur regulon were strongly repressed in response to heme exposure (see below). PerR is known to repress the expression of *fur* (Ledala et al., 2010) and other genes belonging to the PerR regulon, including the catalase-encoding gene *kat* (which is also controlled by SigB), the *fri* gene encoding an iron-binding ferritin-like protein (Rea et al., 2005) and *frvA* which encodes an Fe^2+^ exporter (Pi et al., 2016). Our data indicates that heme exposure may increase the level of H_2_O_2_, which leads to de-repression of these PerR-regulated genes (Duarte and Latour, 2013). In addition, we note that 5 LisR-regulated genes (including *htrA* encoding a serine protease) were among the most up-regulated genes, implying that genes involved in the cell envelope stress response also respond to heme stress, as previously suggested (Dos Santos et al., 2018) (Figure 1B). For example, the LisR-regulated gene *lmo2210*, which is induced by ethanol and cefuroxime (Gottschalk et al., 2008; Nielsen et al., 2012), also relied on LisR for its induction, when *L. monocytogenes* is exposed to heme stress (Supplementary Figure S2). Finally, it should be noted that the 2nd and 3rd most up-regulated genes (*lmo2580* and *lmo2581*) correspond to the predicted HssRS-regulated heme-efflux system *hrtAB*, suggesting that *L. monocytogenes* holds an inducible heme detoxification system regulated by the same homologous TCS as seen in other Gram-positive bacteria (Torres et al., 2007) (Figure 1B). The role of HrtAB and HssRS in *L. monocytogenes* will be further addressed below.

### Heme Exposure Leads to Repression of Genes Involved in Iron/Heme Uptake and Utilization

When analyzing the 297 down-regulated genes with a 4.0-fold cutoff, we observed that a large proportion belonged to the Fur regulon (21 genes; see Figure 1C). Notably, of the 10 most down-regulated genes, half belonged to the Fur regulon (Supplementary Table S3). In this case, the down-regulated genes were only Fur-dependent, and not regulated by PerR. Generally, Fur functions as a Fe^2+^-dependent repressor and Fur-regulated genes code mainly for proteins involved in iron/heme uptake and utilization, such as the heme acquisition (*lmo2186-lmo2180*) and heme transport (*lmo2429-lmo2430*) operons, ferrous iron transport systems (*lmo0365-lmo0367* and *lmo2104-lmo2105*), the iron uptake regulator and ferrichrome transport system (*lmo1959-lmo1957*), ABC transporters (*lmo1960-lmo1961* and *lmo1131-lmo1132*), and the monocistronic gene *lmo0484* (McLaughlin et al., 2012). The latter, encoding a putative heme oxygenase, as well as *lmo2185* and *lmo2186* encoding two heme-binding proteins, were recently found to be highly repressed in the presence of excess heme (Dos Santos et al., 2018). Indeed, *lmo2185* and *lmo2186* correspond to the 1st and 3rd most down-regulated genes in the present study (Supplementary Table S3). Altogether, these findings demonstrate that *L. monocytogenes* reacts to heme stress by efficiently shutting down the expression of Fur-regulated genes involved in iron/heme uptake and utilization.

The second most represented regulon was CodY (19 down-regulated genes; Figure 1C). CodY is a transcriptional regulator that responds to GTP and branched chain amino acids, and it generally serves to control the transition from conditions that allow exponential growth to conditions that limit growth (Bennett et al., 2007). Notably, 12 genes belonging to the CodY regulon are also known to be down-regulated under oxygen-limiting growth conditions (Supplementary Table S3), including the 2nd most down-regulated gene, *lmo1999*, which encodes a glucosamine-fructose-6-phosphate aminotransferase (Supplementary Table S3). As stated above, these findings support that heme exposure promotes a switch from aerobic to anaerobic metabolism.

### The *hrtAB* Genes, Predicted to Encode a Heme Efflux Pump, Are Highly Expressed Upon Heme Exposure in a HssR-Dependent Manner

**FIGURE 2:**
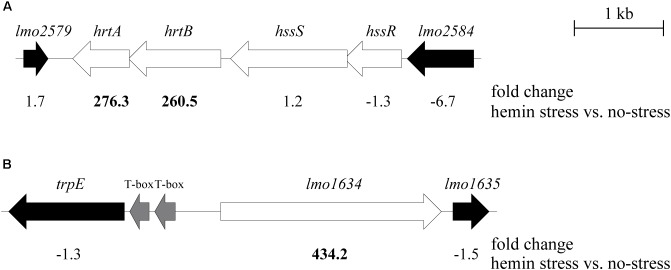
Genomic overview of the three most up-regulated genes in *L. monocytogenes* upon heme exposure. Physical maps of the **(A)** *hrtAB*, *hssRS*, and **(B)** *lmo1634* gene regions in strains EGD-e and LO28; the arrows indicate gene orientation. White arrows represent genes of interest, black arrows flanking genes and the gray arrows T-boxes. The numbers shown underneath each gene are the fold changes obtained from the RNA-seq analysis of the transcripts in EGD-e cells stressed with hemin, compared to the control condition. The size of the genes is drawn to scale.

**FIGURE 3:**
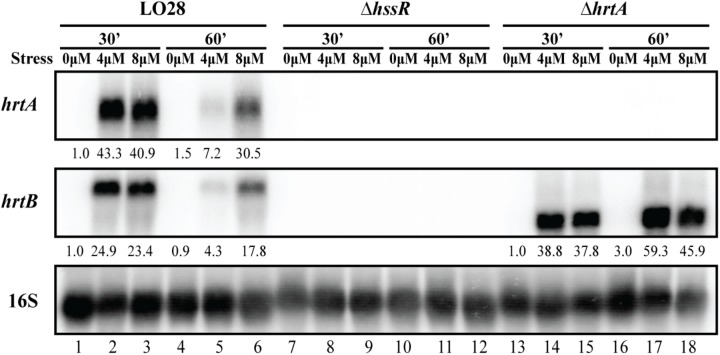
Transcriptional analysis of *hrtAB* expression during heme stress. Samples were taken from LO28 wild-type, Δ*hssR* and Δ*hrtA* cultures exposed to 4 or 8 μM hemin stress for 30 and 60 min, as well as from non-stressed cultures (0 μM). The northern blot was probed for *hrtA* mRNA, *hrtB* mRNA and 16S rRNA (loading control). Levels of *hrtA* mRNA and *hrtB* mRNA (normalized to 16S) relative to the ‘0 μM, 30 min’ sample of each strain are shown below each lane.

As mentioned, the 2nd and 3rd most up-regulated genes in our study were *lmo2580* (*hrtA*; 276-fold) and *lmo2581* (*hrtB*; 260-fold), respectively, predicted to encode HrtAB (Torres et al., 2007). Northern blot analysis confirmed that *hrtA* is indeed highly upregulated in EGD-e upon heme exposure (Supplementary Figure S1). HrtAB was first described in *S. aureus* as an efflux pump, which was highly up-regulated upon exposure to exogenous heme (Friedman et al., 2006). The heme-regulated transporter was later shown to be activated by the heme sensor system, HssRS, which responds to heme exposure (Torres et al., 2007). The same study also predicted the presence of both the HssRS and HrtAB systems in *L. monocytogenes* and other Gram-positive species (Torres et al., 2007). Indeed, in the genome of *L. monocytogenes* EGD-e, the *hssRS* operon is placed upstream of the *hrtAB* operon (Figure 2A), suggesting a regulatory link between *hrtAB* and the HssRS TCS. Notably, the *hssRS* and *hrtAB* operons are organized in the same manner in *L. monocytogenes* strain LO28, which was used in a recent study demonstrating a role for the LisRK TCS and LhrC sRNAs in the response of *L. monocytogenes* to heme toxicity (Dos Santos et al., 2018) (Figure 2A). Importantly, the *hrtA* and *hrtB* genes in *L. monocytogenes* LO28 were also highly induced by heme stress (Figure 3; described below). To investigate the function of these systems in *L. monocytogenes*, two deletion mutants were constructed in LO28: a strain lacking the response regulator gene, *hssR*, and a strain deleted for *hrtA*, encoding the ATPase of the HrtAB efflux pump. To confirm the induction of *hrtAB* in response to heme exposure, as well as the HssR-dependency and co-transcription, the *hrtAB* mRNA levels were determined via northern blot analysis on total RNA purified from *L. monocytogenes* LO28 wild-type, Δ*hssR* and Δ*hrtA* cells subjected to 4 or 8 μM hemin for 30 and 60 min; non-stressed samples were used as control. As seen in Figure 3, *hrtA* and *hrtB* levels were clearly induced in wild-type cells with both hemin concentrations (4 and 8 μM) after 30 min of stress, when compared to non-stressed samples at the same time point. After 60 min, the wild-type strain seemed to have adapted to 4 μM hemin (levels close to the ones detected in non-stressed cells), whereas in the case of 8 μM hemin, the expression decreased compared to the 30 min time point, however, still greatly detectable. Additionally, both *hrtA* and *hrtB* showed no detectable expression in the Δ*hssR* strain, confirming the HssR-dependent expression in response to heme stress. As for the Δ*hrtA* strain, the bands corresponding to *hrtB* match the size of *hrtB* alone, while the bands detected in the wild-type strain correspond to the size of *hrtAB*, meaning that indeed the genes are co-transcribed. It is also noticeable that the *hrtB* levels increased over time in the Δ*hrtA* background, opposite to what happened in the wild-type strain, suggesting that the lack of *hrtA* leads to a non-functional efflux pump, resulting in no alleviation of the toxic effects of heme and a constant expression of the detoxification system in the Δ*hrtA* mutant strain.

**FIGURE 4:**
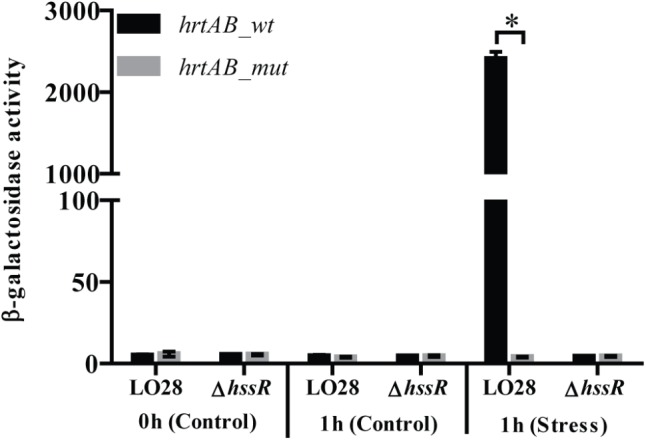
Transcriptional analysis of the *hrtAB* promoter. Plasmids containing the wild-type or mutated version of *hrtAB* promoter region fused to *lacZ* were transformed into LO28 wild-type and Δ*hssR*. The resulting strains were grown up to OD_600_ = 0.35 and stressed with hemin (8 μM), after control samples had been taken (0 h, Control). Further samples for a following β-galactosidase assay were withdrawn after 1 h (1 h, Control and Stress). Results are the average of three biological replicates, each carried out in technical duplicates. After 1 h of heme stress, a significant difference between the LO28 wild-type strain and the Δ*hssR* mutant carrying the *hrtAB* wild-type promoter was observed (^∗^*p* < 0.0001).

To further investigate the induction of *hrtAB* by heme, we aimed to determine the promoter activity of the operon using transcriptional fusions of the promoter to the reporter gene *lacZ* in the vector pTCV-lac (Poyart and Trieu-Cuot, 1997). First, primer extension was performed to determine the transcription start site of *hrtAB* (Supplementary Figures S3A,B). Notably, the *hrtAB* promoter region contains a direct repeat corresponding to the predicted HssR binding site (Supplementary Figure S3A) (Stauff et al., 2007). To confirm the importance of this direct repeat for HssR-dependent activation of *hrtAB*, a mutated version of the promoter was created, where four separate nucleotides spread throughout the repeat region were mutated, as done in studies of the HssR box in *S. aureus* (Supplementary Figure S3A and Supplementary Table S1) (Stauff et al., 2007). Both wild-type (pTCV-*hrtAB_wt*-*lacZ*) and mutated (pTCV-*hrtAB_mut*-*lacZ*) versions of the *hrtAB* promoter fused to *lacZ* were transformed into LO28 wild-type and Δ*hssR* cells. The β-galactosidase activity was determined 1 h after exposing the cultures to hemin (8 μM) and non-stressed cultures were included as controls (Figure 4). After 1 h of heme stress, a massive increase in the β-galactosidase activity was observed in the wild-type strain carrying the pTCV-*hrtAB_wt*-*lacZ* fusion plasmid, while no increase in activity was detected in the Δ*hssR* cells harboring the same plasmid. On the other hand, the cells carrying the pTCV-*hrtAB_mut*-*lacZ* fusion plasmid did not produce any increase in the β-galactosidase activity. Overall, the results confirmed the induction of the *hrtAB* promoter by heme exposure and the requirement of both HssR and an intact repeat region for induction of *hrtAB* expression when *L. monocytogenes* faces excess concentrations of heme.

### HrtA and HssR Are Essential for Growth of *L. monocytogenes* Under Heme Stress Conditions

**FIGURE 5:**
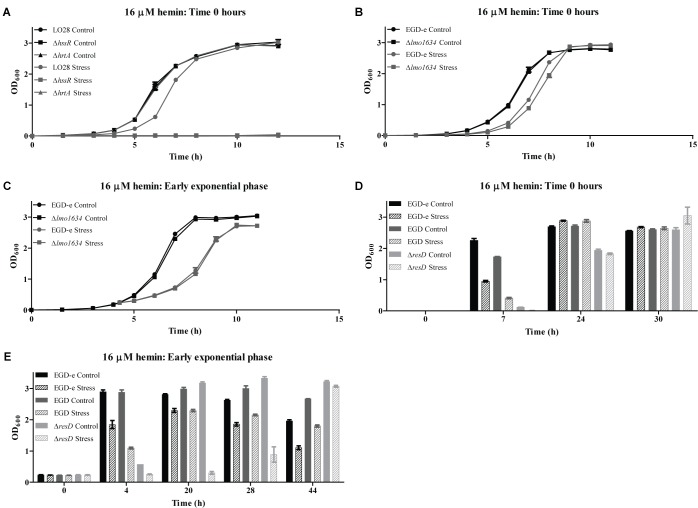
Stress tolerance assay. **(A)** LO28 wild-type, Δ*hssR* and Δ*hrtA* strains were grown in BHI (Control) and BHI containing 16 μM hemin (Stress). **(B)** EGD-e wild-type and Δ*lmo1634* strains were grown in BHI (Control) and BHI containing 16 μM hemin (Stress). **(C)** EGD-e wild-type and Δ*lmo1634* were grown in BHI to OD_600_ = 0.2. Then, the cultures were split in two; one half was stressed with 16 μM hemin (Stress) and the other half kept unstressed (Control). **(D)** Wild-type EGD-e, wild-type EGD and EGDΔ*resD* were grown in BHI (Control) and BHI containing 16 μM hemin (Stress). **(E)** Wild-type EGD-e, wild-type EGD and EGDΔ*resD* were grown in BHI to OD_600_ = 0.2. Then, the cultures were split in two; one half was stressed with 16 μM hemin (Stress) and the other half kept unstressed (Control). **(A–C)** Bacterial growth was monitored until the cultures reached stationary phase, or **(D,E)** punctual OD_600_ measurement were taken at the indicated time points. For each assay, the average of three biological replicates and respective standard deviations are shown.

To investigate the role of the predicted heme detoxification system in heme tolerance, a growth assay was performed (Figure 5A), where the growth of the wild-type LO28 strain and strains lacking *hrtA* or *hssR* was compared when these cultures were exposed to 16 μM hemin. No difference in growth was observed between the wild-type and the two mutant strains when grown under control conditions. However, when hemin was added to the growth medium, the growth of all strains was affected. For the wild-type culture, the cells required a longer time to adapt to the new stress condition, but reached a final OD_600_ value comparable to the non-stressed culture. As for the mutant strains, neither of the two could grow in the presence of 16 μM hemin, showing that a functional TCS and consequently a functional efflux pump are essential for growth when *L. monocytogenes* faces high concentrations of heme. Collectively, these findings correspond well with the predicted function of HssRS/HrtAB as a highly conserved heme detoxification system in Gram-positive bacteria (Stauff et al., 2007).

### LAP: A Novel Player in the Response of *L. monocytogenes* to Excess Heme?

**FIGURE 6:**
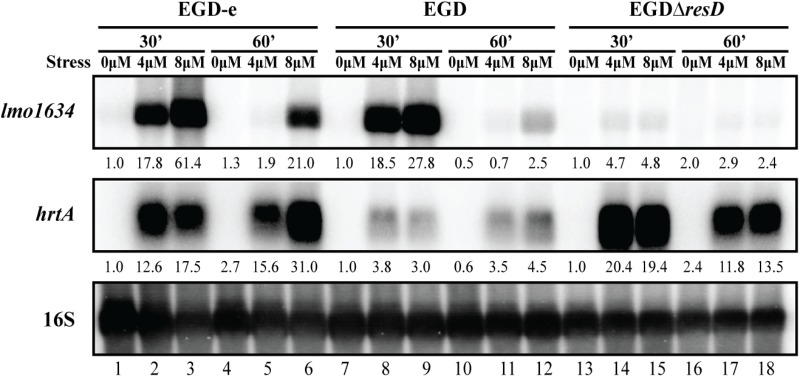
Transcriptional analysis of *lmo1634* expression during heme stress. Samples were taken from wild-type EGD-e, wild-type EGD and mutant EGDΔ*resD* cultures exposed to 4 or 8 μM hemin stress for 30 and 60 min, as well as from non-stressed cultures (0 μM). Northern blots were probed for *lmo1634* mRNA, *hrtA* mRNA and 16S rRNA (loading control). Levels of *lmo1634* mRNA and *hrtA* mRNA (normalized to 16S) relative to the ‘0 μM, 30 min’ sample of each strain are shown below each lane.

As mentioned above, the most up-regulated gene in response to heme exposure was *lmo1634* (Figure 2B and Supplementary Table S3). Northern blot analysis confirmed that *lmo1634* is indeed highly upregulated in EGD-e upon heme exposure (Supplementary Figure S1). The *lmo1634* gene encodes the *Listeria* adhesion protein (LAP), a putative bifunctional acetaldehyde-CoA/alcohol dehydrogenase (Jaradat et al., 2003) that interacts with the host-cell receptor Hsp60 (Wampler et al., 2004). Interestingly, LAP was recently shown to promote translocation of *L. monocytogenes* across the intestinal barrier *in vivo* (Drolia et al., 2018). A Protein Blast revealed that LAP is highly identical to the Aldehyde-alcohol dehydrogenase, AdhE, from *B. subtilis* (74%) and *S. aureus* (65%). In *S. aureus*, *adhE* was shown to be part of the SrrAB regulon, a TCS important for the resistance of the bacterium to nitrosative stress (Kinkel et al., 2013), which is also involved in growth under anaerobic conditions (Throup et al., 2001). This TCS is homologous to ResDE in *L. monocytogenes* (Larsen et al., 2006). The response regulator ResD is known to control respiration and sugar uptake in *L. monocytogenes* strain EGD and is required for virulence gene repression in response to several types of sugars (Larsen et al., 2006). Although ResD contributes to cellular invasion *in vitro*, no influence of ResD was observed in a murine infection model (Larsen et al., 2006). Based on this information, we aimed to investigate the potential effect of ResD on *lmo1634* by using northern blot analysis (Figure 6). For this experiment, total RNA was harvested from the *resD* deletion mutant, EGDΔ*resD*, constructed by Larsen et al. (2006) together with the parental strain EGD, after exposing the cells to heme stress. For comparison, the EGD-e wild-type strain, which was used for the RNA-seq analysis, was included in the experiment (Figure 6). Interestingly, the induction of *lmo1634* followed the same pattern observed for *hrtAB*: high levels of the mRNA after 30 min of 4 and 8 μM hemin exposure for both wild-type strains (EGD-e and EGD), and after 60 min the cells seemed to have adapted to 4 μM hemin and showed reduced levels of induction with 8 μM hemin compared to the 30 min time point. Regarding *lmo1634* mRNA levels in the Δ*resD* background, only a slight increase was seen for both concentrations of hemin when compared to the parental strain (EGD). As a control, we probed for the *hrtA* mRNA (Figure 6). We note that *hrtA* is highly induced in the EGDΔ*resD* strain; even more than in the corresponding wild-type strain (EGD). To investigate if the TCS HssRS could be involved in controlling the transcription of *lmo1634*, a northern blot analysis was performed using RNA purified from heme-stressed *L. monocytogenes* LO28 wild-type and Δ*hssR* strains (Supplementary Figure S4). We found that *lmo1634* was equally induced in the LO28 wild-type and Δ*hssR* strains, demonstrating that HssR is not required for heme-induced transcription of *lmo1634* (Supplementary Figure S4). In line with this result, no obvious binding sequence for HssR could be found in the promoter region of *lmo1634*.

Altogether, these results demonstrated a strong up-regulation of *lmo1634* upon heme exposure of three wild-type strains of *L. monocytogenes* (EGD-e, EGD, and LO28). Furthermore, our data suggest a clear dependence on ResD, but not HssR, for heme-inducible expression of *lmo1634*. The residual induction in the *resD* mutant background indicates that other regulators may be involved as well.

### LAP Is Not Essential for Growth Under Heme Stress Conditions

To investigate the role of *lmo1634* in the prevention of heme toxicity, an in-frame deletion mutant was constructed in the EGD-e background, and the growth of both the wild-type and mutant strain was followed upon addition of 16 μM hemin to the growth medium (Figure 5B). For both strains, growth was affected by the addition of hemin; however, no major difference in growth between the mutant and wild-type strain was observed (Figure 5B). Hence, another approach was attempted, where 16 μM hemin was added to exponentially growing cells (OD_600_ = 0.2) (Figure 5C). Similarly, no significant differences were observed between the two strains, suggesting that although *lmo1634* is highly induced upon heme exposure, the gene does not contribute to growth of *L. monocytogenes* in heme-rich environments. As our results suggested that *lmo1634* is at least partially regulated by ResD in response to heme stress, growth experiments were performed to evaluate the growth of the EGDΔ*resD* strain and its parental strain EGD under excess heme conditions. Again, the EGD-e strain, which was used for the RNA-seq analysis, was included for comparison. In these experiments, punctual OD_600_ measurements were taken during 30 or 44 h of growth, when hemin was added to the cultures from the beginning of the growth experiment (Figure 5D), or to exponentially growing cells (Figure 5E), respectively. We note, that growth of the two wild-type strains (EGD-e and EGD) was comparable under all conditions tested. As previously observed, the EGDΔ*resD* strain presented an impaired growth compared to the EGD wild-type under non-stressed conditions (Larsen et al., 2006). Likewise, under heme stress conditions, growth of the EGDΔ*resD* strain was impaired in comparison to the EGD wild-type, however, the mutant could clearly adapt, reaching final OD_600_ values comparable to the EGD wild-type. Collectively, these results indicate that LAP and ResD are not required for *L. monocytogenes* to resist heme toxicity.

## Discussion

Bacterial pathogens are known to utilize heme as an iron source during infection, however, the high levels of heme encountered by the pathogens in the bloodstream or blood-rich organs may become toxic to them (Choby and Skaar, 2016; Joubert et al., 2017). Under aerobic conditions, H_2_O_2_ reacts with Fe^2+^ liberated from heme, which generates toxic hydroxyl radicals and Fe^3+^ via Fenton chemistry (Imlay, 2013). Cellular reductants such as NADH will reduce Fe^3+^ to Fe^2+^, allowing the recycling of iron and further production of reactive oxygen species (ROS) (Brumaghim et al., 2003). Ultimately, heme-induced production of ROS may lead to oxidative damage of DNA, lipids and proteins (Nagababu and Rifkind, 2004). Very little is known about how *L. monocytogenes* avoids heme-mediated toxicity, and thus our study focused on understanding how the transcriptome of this pathogenic bacterium is affected upon exposure to excess heme. Our findings suggest that the response of *L. monocytogenes* to heme exposure is multifaceted. Firstly, we observed that *L. monocytogenes* reacts to heme stress conditions by inducing the SOS response. Usually, the SOS response is activated when single-stranded DNA accumulates in the cell. This situation results in the activation of RecA, which in turn stimulates cleavage of LexA and, ultimately, the induction of the SOS response (Schlacher et al., 2006). Induction of the entire LexA/RecA regulon demonstrates that heme exposure indeed leads to DNA damage, which requires an immediate DNA repair. The DNA damage observed upon heme exposure is most likely caused by heme-induced production of ROS. Indeed, the transcriptome analysis revealed the induction of several genes involved in dealing with oxidative stress. For example, the induction of Fur/PerR- and SigB-regulated *kat* (16.7-fold) indicates that oxidative stress is a major component of the heme-induced toxicity (Mongkolsuk and Helmann, 2002). Usually, catalase (encoded by *kat*) and superoxide dismutase (SOD) work together in detoxification of ROS, as SOD converts superoxide anions to H_2_O_2_, and then catalase converts H_2_O_2_ into water and oxygen (Dallmier and Martin, 1988; Azizoglu and Kathariou, 2010). Indeed, *sod* is induced by heme exposure as well (2.3-fold; Supplementary Table S2) supporting that these detoxification enzymes are needed for *L. monocytogenes* to deal with the toxic effects of excess heme.

Our study also suggests that *L. monocytogenes* efficiently deals with the iron-overload that likely arises following heme exposure. In line with previous observations (McLaughlin et al., 2012), we found that the Fur/PerR-regulated *frvA* gene was strongly induced (68.2-fold) in response to heme exposure. The *frvA* gene was originally described to encode a heme exporter protecting against heme toxicity (McLaughlin et al., 2012), but more recently it was shown to act as an iron exporter (Pi et al., 2016). Indeed, the gene appeared to be induced by high levels of available iron (Pi et al., 2016) and to be positively regulated by Fur in such conditions (Ledala et al., 2010; Pi et al., 2016), matching the results we obtained in our transcriptomic analysis. Importantly, *frvA* is also required for full virulence in *L. monocytogenes* (McLaughlin et al., 2012, 2013; Pi et al., 2016). The Fur/PerR-regulated gene *fri* was highly induced as well in response to heme stress (10.1-fold). The iron-binding ferritin-like protein Fri (also known as Dps) holds a protective role against peroxide stress and contributes to virulence of *L. monocytogenes* (Dussurget et al., 2005; Olsen et al., 2005). At the transcriptional level, *fri* is up-regulated by iron depletion and it promotes growth under iron-limiting conditions (Polidoro et al., 2002; Olsen et al., 2005), but also favors growth in the presence of iron (Dussurget et al., 2005), even though the transcript abundance decreases under iron-rich conditions (Fiorini et al., 2008). Considering that exposure to excess heme may lead to iron overload, our results suggest that *fri* is induced to provide enough Fri protein for iron storage and thereby avoid iron-mediated generation of ROS. Finally, *L. monocytogenes* reacts to heme exposure by efficiently shutting down the expression of Fur-regulated genes involved in iron uptake. Altogether, *L. monocytogenes* seems to deal with the iron-overload following heme exposure by increasing its capacity to export and store iron, and in parallel it reacts to prevent further uptake of iron.

In line with our previous observations (Dos Santos et al., 2018), we found that *L. monocytogenes* also reacts to heme stress by shutting down the uptake and metabolism of heme itself. Additionally, we demonstrated here that *L. monocytogenes* induces the expression of a putative heme-export system in response to excess heme. The 2nd and 3rd most up-regulated genes, *hrtAB*, were predicted to encode an efflux pump that alleviates excess heme (Torres et al., 2007). Such systems have been widely described in other Gram-positive bacteria (Stauff et al., 2007; Torres et al., 2007; Pedersen et al., 2008; Stauff and Skaar, 2009a; Bibb and Schmitt, 2010; Fernandez et al., 2010; Lechardeur et al., 2012; Sachla et al., 2014; Wakeman et al., 2014; Schmidt et al., 2016; Joubert et al., 2017); however, we were still lacking information on the role of HrtAB in *L. monocytogenes*. Our experiments revealed the importance of *hrtA* to allow the adaptation and growth of *L. monocytogenes* under heme-rich conditions. Furthermore, our results demonstrated that expression of *hrtAB* relies on a functional HssRS TCS, which likely responds to heme exposure. Indeed, a strain lacking *hssR* was incapable of growing in heme-replete medium. The genomic organization of *hssRS* and *hrtAB* resembles the one seen for *Staphylococcus* spp. (Torres et al., 2007), and a conserved sequence in the promoter upstream from *hrtAB*, which is most likely recognized by HssR, is crucial for expression of the efflux system. Based on our findings, we propose that when *L. monocytogenes* faces high concentrations of heme, the molecule is transported into the cell or simply diffuses trough the peptidoglycan layer (Lechowicz and Krawczyk-Balska, 2015). Then, the heme molecule will either enter the cell or, most likely, position itself in the membrane as suggested for *S. aureus* (Skaar et al., 2004; Wakeman et al., 2012) and Group A *Streptococcus* (Sachla et al., 2014), where it will be sensed by the HssS histidine kinase, which in turn activates HssR. The response regulator can then activate transcription from the *hrtAB* promoter. Indeed, results from the northern blot analysis suggested that heme is constantly sensed by HssS: in the Δ*hrtA* background, where heme is not being exported due to a non-functional efflux pump, the *hrtB* transcript is constantly expressed over time, opposite to what happens in the wild-type background, where heme alleviation is expected to occur. Intracellular heme sensing (including membrane-associated heme sensing) has been suggested to take place in other microorganisms (Torres et al., 2007; Fernandez et al., 2010; Lechardeur et al., 2012). Regardless, the mechanism by which HssS in *L. monocytogenes* senses heme (or a byproduct of heme metabolism) still requires further investigation. In fact, the exact compound that is sensed by the heme-responsive systems has been the focus of diverse studies: *L. lactis* HrtR showed high specificity for heme (or FePPIX, where PPIX is short for protoporphyrin IX), but also for GaPPIX and to a lesser extent MnPPIX (Lechardeur et al., 2012); Group A *Streptococcus* PefR was shown to bind heme and PPIX with high affinity (Sachla et al., 2014); and *S. aureus* HssRS senses heme, MnPPIX, GaPPIX and more modestly ZnPPIX, however, HrtAB is ineffective at detoxifying most of these molecules, as only heme is efficiently transported and ZnPPIX is exported to a lesser extent (Wakeman et al., 2014). Further studies on the compound(s) sensed and/or exported by the HssRS and HrtAB systems in *L. monocytogenes* are essential to understand what is indeed causing the heme toxicity experienced by the bacterium, and how we could use that knowledge to develop novel antimicrobial strategies targeting the harmful effects of this pathogen. As a matter of fact, a role for the HssRS and HrtAB systems in virulence in several pathogens has been proposed: in *S. aureus*, the inactivation of the heme responsive systems resulted in enhanced liver-specific virulence (Torres et al., 2007); in *B. anthracis*, HrtAB was shown to be up-regulated in a murine infection model (Stauff and Skaar, 2009a); and in *S. agalactiae*, HrtAB is required for full virulence and survival in the heart, kidney, and liver (Joubert et al., 2017).

The most highly induced gene, *lmo1634*, encodes the *Listeria* adhesion protein (LAP) that promotes bacterial translocation across the intestinal epithelial barrier (Drolia et al., 2018). In addition to its role as an adhesion factor, LAP is predicted to act as a bifunctional acetaldehyde CoA/alcohol dehydrogenase (ADH), which most likely promotes the reduction of acetyl-CoA to acetaldehyde and further to ethanol under anaerobic growth (Muller-Herbst et al., 2014). During this reaction, NADH is re-oxidized to NAD^+^ (Muller-Herbst et al., 2014). LAP shares protein identity with ADH proteins from other species (Kim et al., 2006), including AdhE from *E. coli*, which is induced under anaerobic conditions (Clark and Cronan, 1980; Leonardo et al., 1996). Likewise, *lmo1634* is expressed under anaerobic conditions (Burkholder et al., 2009), but also in response to antibiotic exposure (Knudsen et al., 2016). Sub-lethal concentrations of antibiotics caused a shift from aerobic to anaerobic metabolism (Knudsen et al., 2016), and we noted a similar metabolic shift when exposing *L. monocytogenes* to heme stress (this study). Knudsen et al. (2016) speculated that upon antibiotic exposure, *L. monocytogenes* shifts to anaerobic metabolism and production of ethanol to avoid recycling of NADH to NAD^+^ by respiration and production of ROS. We hypothesize that *L. monocytogenes* employs a similar strategy following heme stress as an attempt to reduce heme-mediated production of ROS. However, despite the increased expression of *lmo1634* in response to antibiotics or excess heme, deletion of *lmo1634* did not alter the susceptibility of *L. monocytogenes* toward these stressors [(Knudsen et al., 2016); this study]. Likewise, disruption of *lmo1634* did not prevent *L. monocytogenes* from growing under anaerobic conditions (Muller-Herbst et al., 2014). These results could be due to redundancy between *lmo1634* and other genes with similar functions in *L. monocytogenes* (Muller-Herbst et al., 2014; Knudsen et al., 2016). A role for the response regulator ResD in preventing heme toxicity was rejected as well, although ResD clearly contributes to heme-dependent induction of *lmo1634*. Alternatively, heme-mediated induction of *lmo1634* might be linked to its function as an adhesion factor. We speculate that heme could act as a host-derived signal to induce the expression of LAP in blood-rich environments. So far, LAP has been implicated in the translocation of *L. monocytogenes* across the intestinal barrier; however, LAP is also highly produced by *L. monocytogenes* in blood, relative to bacteria grown in rich medium, suggesting that LAP could play a role in the dissemination of *L. monocytogenes* to deep organs (Quereda et al., 2016). Although cell adhesion studies and mouse bioassays clearly support a major role for LAP during the intestinal phase of infection, a LAP-deficient strain also showed reduced adhesion to Vero kidney cells (Jaradat et al., 2003). Interestingly, after intraperitoneal infection of mice, a LAP-deficient mutant translocated less efficiently to the liver, compared to the wild-type (approximately 1 log difference) (Jaradat et al., 2003), supporting that LAP might play a role at the later stages of infection.

In addition to *lmo1634*, other genes with important roles in virulence were found to be highly up-regulated upon heme exposure. The most obvious example was *prfA* encoding the key virulence regulator in *L. monocytogenes* (6.7-fold of induction; Supplementary Table S3) (de las Heras et al., 2011). Notably, *prfA* and genes belonging to the PrfA-regulon are highly induced when *L. monocytogenes* is exposed to whole human blood (Toledo-Arana et al., 2009). Even though the transcription of *prfA* was clearly induced under heme stress, the PrfA-regulon was not represented in our study, most likely because the activity of PrfA was repressed during growth in the BHI medium used in our experiments (Ripio et al., 1996). Still, transcriptional induction of *prfA* suggests an activation of virulence genes upon sensing of excess heme. This hypothesis was further supported by the observed induction of other virulence-associated genes, such as the gene *lmo1800* encoding for the protein *Listeria* phosphatase A (LipA; 11.5-fold), which was shown to display phosphatase activity on both protein and lipid substrates and to play a major role in the virulence of *L. monocytogenes* in mice (Kastner et al., 2011). Likewise, LmiA, a LPXTG surface protein encoded by *lmo1413* (5.6-fold), which is absent in non-pathogenic *Listeria*, was shown to play a role as an auxiliary invasin promoting bacterial entry into host cells (Mariscotti et al., 2014). In addition, transposon insertions into *lmo0964* (4.1-fold) and into *lmo2460* (5.5-fold) revealed a hemolytic defect of these two mutants, suggesting a possible role in controlling the production, activity, or secretion of listeriolysin O (Zemansky et al., 2009). In the same way, *aroE* (9.9-fold), encoding 5-enolpyruvylshikimate-3-phosphate synthase, contributes to virulence of *L. monocytogenes* (Stritzker et al., 2004), supporting that mutations in the basic branch of the aromatic amino acid biosynthesis pathway of *L. monocytogenes* efficiently attenuate virulence, as seen for other bacterial pathogens as well (Stritzker et al., 2004). Finally, we noted that a clear majority of the heme-induced genes codes for hypothetical proteins; intriguingly, several of those genes were found to be up-regulated in whole human blood as well (Toledo-Arana et al., 2009) (Supplementary Table S3). Thus, multiple proteins with putative roles in the adaptation of *L. monocytogenes* to blood-rich host environments remain to be characterized.

To summarize, we found that *L. monocytogenes* employs an array of genes when dealing with heme toxicity. Genes contributing to various stress responses in *L. monocytogenes*, such as the SOS response, were highly induced upon heme exposure, whereas other genes, such as those encoding iron/heme uptake systems, were instantly repressed. As predicted, our study supported that the HssRS-regulated heme exporter *hrtAB* is required for *L. monocytogenes* to avoid heme toxicity. However, a role for the most up-regulated gene, *lmo1634*, in dealing with heme toxicity, could not be disclosed. Based on this finding, we speculate that *L. monocytogenes* perceives heme as a signaling molecule to control expression of the LAP adhesion protein encoded from *lmo1634*, as well as other virulence factors, including the key virulence regulator PrfA. Future studies should aim at defining the molecular mechanism by which *L. monocytogenes* uses heme to sense the host environment and regulate virulence genes during infection.

## Author Contributions

PS and BK conceived and designed the experiments. PS, PL, PM-G, and EL performed the experiments. PS, PL, EL, and BK analyzed the data. PS and BK wrote the paper. All authors read and approved the final manuscript.

## Conflict of Interest Statement

The authors declare that the research was conducted in the absence of any commercial or financial relationships that could be construed as a potential conflict of interest.
