# Supplementary material for: Listeria monocytogenes Relies on the Heme-Regulated Transporter hrtAB to Resist Heme Toxicity and Uses Heme as a Signal to Induce Transcription of lmo1634, Encoding Listeria Adhesion Protein
Source: Front Microbiol. 2018 Dec 18;9:3090. doi: 10.3389/fmicb.2018.03090 (PMC6305404; doi:10.3389/fmicb.2018.03090)
Supplement: Supplementary file 1 [file Table_1.DOCX]

Supplementary Material

***Listeria monocytogenes* Relies on the Heme-regulated Transporter *hrtAB* to Resist Heme Toxicity and Uses Heme as a Signal to Induce Transcription of *lmo1634*, encoding *Listeria* Adhesion Protein**

Patrícia Teixeira dos Santos^1^, Pernille Tholund Larsen^1^, Pilar Menendez-Gil^1^, Eva Maria Sternkopf Lillebæk^1^, Birgitte Haahr Kallipolitis^1*^

^1^ Department of Biochemistry and Molecular Biology, University of Southern Denmark, Odense M, Denmark

*** Correspondence:**Birgitte H. Kallipolitis
bhk@bmb.sdu.dk

Content:

Supplementary Figure S1, S2, S3 and S4

Supplementary Table S1

**
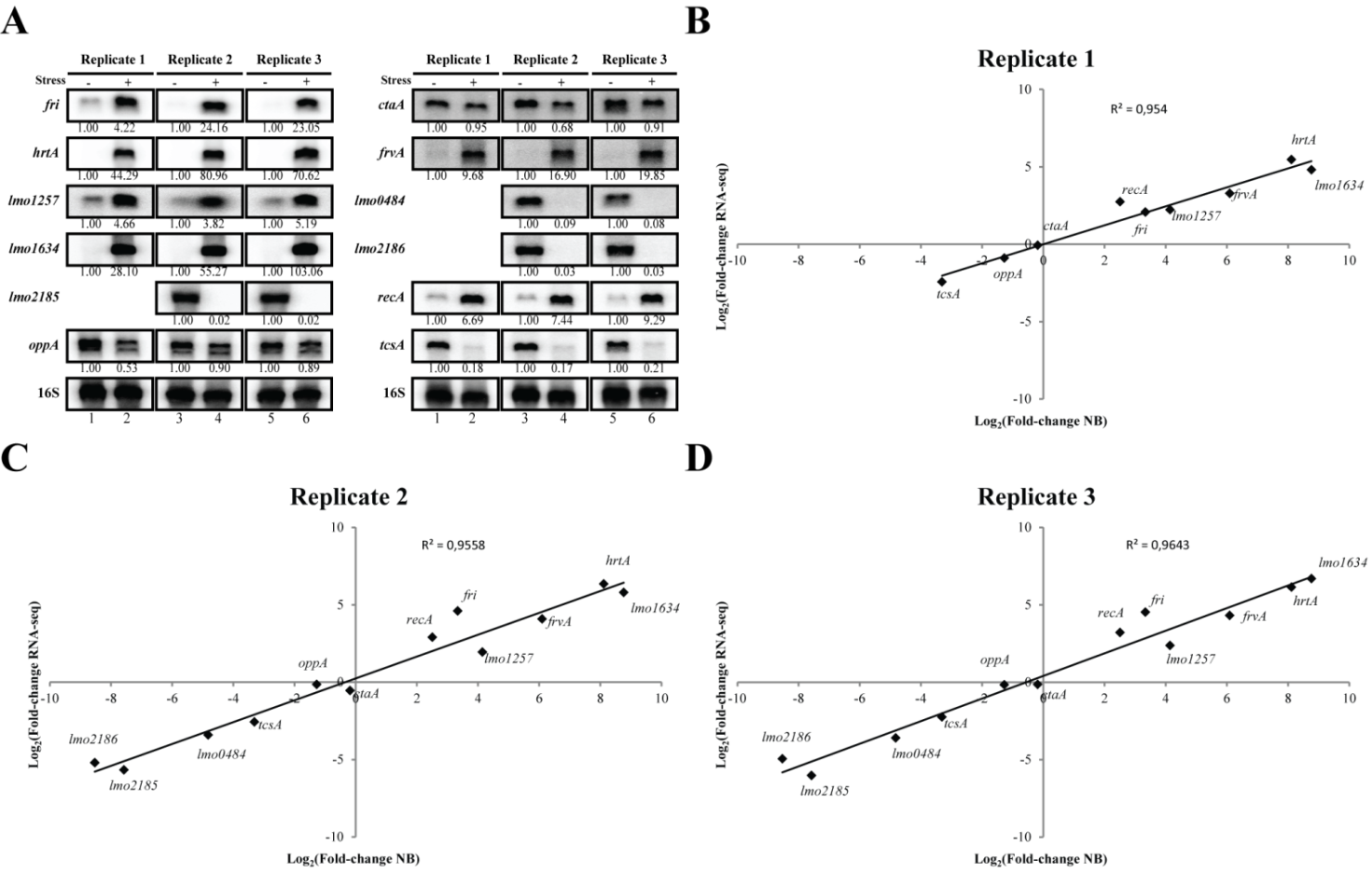
**

**Supplementary Figure S1:** Validation of the RNA sequencing data by northern blot analysis. (**A**) Northern blot analysis of the expression of up to 12 selected mRNAs. Samples were taken from EGD-e wild-type culture exposed to 8 μM hemin stress for 30 min (+), as well as from non-stressed cultures (-). Northern blots were probed for the 12 selected mRNAs and 16S rRNA (loading control). Relative levels of the mRNAs (normalized to 16S) are shown below each lane. The experiment was performed with 2 or 3 biological replicates for each mRNA. (**B**) Comparison of the log_2_ of the fold changes obtained in the RNA-seq and in the northern blot analysis for all 3 biological replicates.

**Supplementary Figure S2:** Transcriptional reporter gene fusion of *lmo2210* promoter. Plasmid containing *lmo2210* promoter region fused to *lacZ* was transformed into LO28 wild-type and Δ*lisR*. The resulting strains were grown up to OD_600_ = 0.35 and stressed with hemin (8 μM), after control samples had been taken (Control). Further samples for a following β-galactosidase assay were withdrawn after 2 hours (Stress). Results are the average of three biological replicates, each carried out in technical duplicates. After 2 hours of stress, a significant difference between the mutant and wild-type cells was observed (**p* < 0.01, ***p* < 0.0001).

**
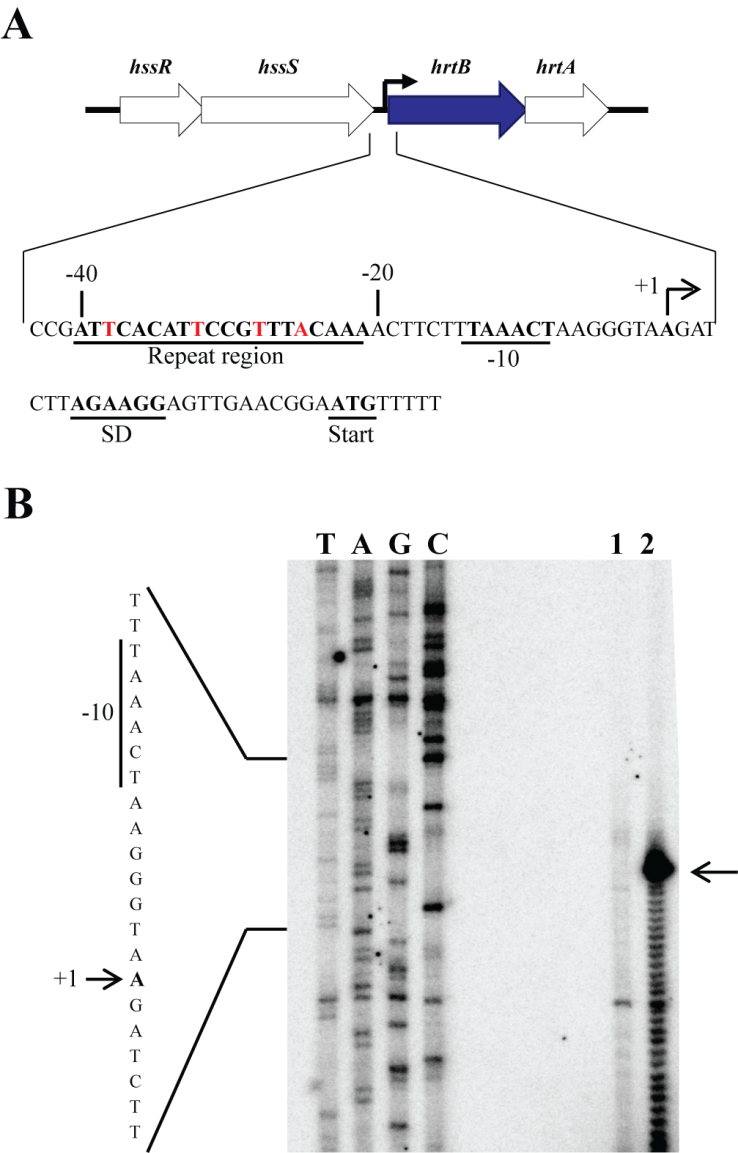
**

**Supplementary Figure S3:** Mapping the transcriptional start site of *hrtAB*. (**A**) Illustration of the genomic location and promoter region sequence for *hrtB*, where *hrtAB* genes are co-transcribed. Transcriptional start sites (+1) are marked by an arrow. The repeat region, -10 box, the predicted SD sequence and the start codon are marked in bold, and the mutated nucleotides are marked in red. (**B**) Primer extension analysis to determine the 5´-end of *hrtB* mRNA. Total RNA from LO28 grown to OD600 = 0.35 and subjected to 8 µM hemin stress (2) or kept unstressed (1) for 1 hour was used for the experiment. T, A, G and C lanes represent the sequencing ladders. The transcriptional start site (+1) is marked in bold and the -10 box is indicated.


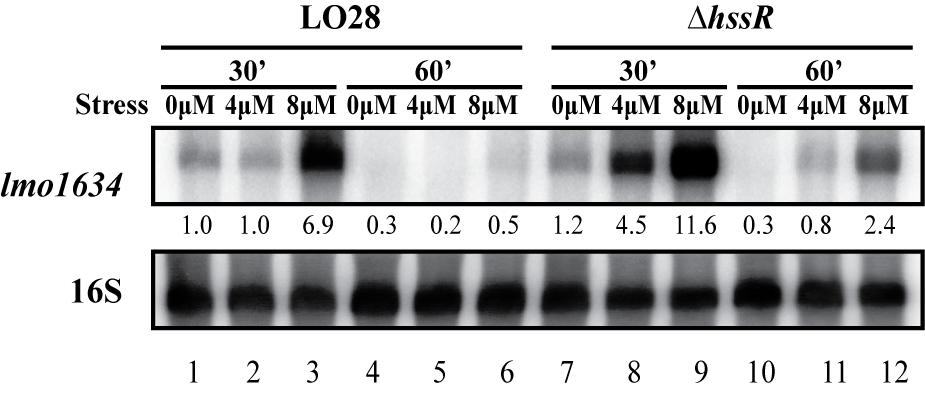


**Supplementary Figure S4:** Transcriptional analysis of *lmo1634* expression during heme stress. Samples were taken from LO28 wild-type and Δ*hssR* cultures exposed to 4 or 8 μM hemin stress for 30 and 60 min, as well as from non-stressed cultures (0 μM). The northern blot was probed for *lmo1634* mRNA and 16S rRNA (loading control). Levels of *lmo1634* mRNA (normalized to 16S) relative to the ‘0 μM, 30 min, LO28’ sample are shown below each lane.

**Supplementary Table S1:** Primers used in this study

| Name | Sequence (5’→ 3’) | Further information |
| --- | --- | --- |
| Cloning for in-frame deletion | | |
| hssR-1 | GGGGGAATTCCTTCGGAAATCCTCGTTAAAACAG | Forward primer for upstream flanking region of *hssR*. EcoRI restriction enzyme site (underlined). |
| hssR-2 | CAAGTTTAAGTATATGCCGATCATC | Reverse primer for upstream flanking region of *hssR*. |
| hssR-3 | **GATCGGCATATACTTAAACTTG**GATGGTA TTCGAATTGTTACGGTG | Forward primer for downstream flanking region of *hssR*. Anneals with hssR-2 (bold). |
| hssR-4 | CCCCGGATCCAGTTCGCCCACATTTTCCAAG | Reverse primer for downstream flanking region of *hssR*. BamHI restriction enzyme site (underlined). |
| hssR-5 | CAGCGGAAATAATATACAGCAG | Forward primer for verification of chromosomal deletion of *hssR*. |
| hssR-6 | GAGTCAATTCCATGATAAGTATCTC | Reverse primer for verification of chromosomal deletion of *hssR*. |
| hrtA-1 | GGGGGAGCTCGAAGCATCGTTACCCAAGTTG | Forward primer for upstream flanking region of *hrtA*. SacI restriction enzyme site (underlined). |
| hrtA-2 | TCGATGACTTGCTCGCCATC | Reverse primer for upstream flanking region of *hrtA*. |
| hrtA-3 | **GATGGCGAGCAAGTCATCGA**GACTTAGT TGACCGCGTAATTC | Forward primer for downstream flanking region of *hrtA*. Anneals with hrtA-2 (bold). |
| hrtA-4 | CCCCGGATCCCAAGAAAGCTTTTTCATCTTCATATTAG | Reverse primer for downstream flanking region of *hrtA*. BamHI restriction enzyme site (underlined). |
| hrtA-5 | GTTATCGCCGCATTTGTTCTAG | Forward primer for verification of chromosomal deletion of *hrtA*. |
| hrtA-6 | ATAACGGGACAGAGCATTATAAAC | Reverse primer for verification of chromosomal deletion of *hrtA*. |
| lmo1634-1 | CCCCGAATTCTGAACACTAACTTCCTTAAAAACCC | Forward primer for upstream flanking region of *lmo1634*. EcoRI restriction enzyme site (underlined). |
| lmo1634-2 | TGCTTTTTGTCCATTGTCTGCT | Reverse primer for upstream flanking region of *lmo1634*. |
| lmo1634-3 | **AGCAGACAATGGACAAAAAGCA**ACAAAGGTGTTTGAAACTAGCTAACTT | Forward primer for downstream flanking region of *lmo1634*. Anneals with lmo1634-2 (bold). |
| lmo1634-4 | CCCCGGATCC AGCCTGTACAAATTCTGCCTC | Reverse primer for downstream flanking region of *lmo1634*. BamHI restriction enzyme site (underlined). |
| lmo1634-5 | CACTTCAGAAAAAGCATGCTA | Forward primer for verification of chromosomal deletion of *lmo1634*. |
| lmo1634-6 | CAAAATGTATTTTGAATCGGAA | Reverse primer for verification of chromosomal deletion of *lmo1634*. |
| pAUL-1 | ATGATTACCGCCCAAGCTTG | Forward primer for verification of plasmid construct |
| pAUL-2 | CAGGACGTTGTAAAACGACG | Reverse primer for verification of plasmid construct |
| *lacZ* fusions | | |
| Fw_*hrtAB* promoter | GGGGGAATTCCCAAAAAACTAAATTTGTAAAATCCGATTCACATTCCGTTTACAAAAC | Forward primer, transcriptional fusion of *hrtAB* promoter in pTCV-lac. EcoRI restriction enzyme site (underlined). |
| Rv_*hrtAB* promoter | CCCCGGATCCCGTAATTTTGCGTGTTTTAATTCTCTTAATGCC | Reverse primer, transcriptional fusion of *hrtAB* promoter in pTCV-lac. BamHI restriction enzyme site (underlined). |
| Fw_*hrtAB* promoter_Mut | GGGGGAATTCCCAAAAAACTAAATTTGTAAAATCCGAT**A**CACAT**A**CCG**A**TT**T**CAAAACTTCTTTAAACTAAG | Forward primer, transcriptional fusion of *hrtAB* promoter with point mutations (bold) in pTCV-lac. EcoRI restriction enzyme site (underlined). |
| Vlac-1 | GTTGAATAACACTTATTCCTATC | Flanking forward primer to check insertions in pTCV-lac. |
| Vlac-2 | CTTCCACAGTAGTTACACCACC | Flanking reverse primer to check insertions in pTCV-lac. |
| Primer extension | | |
| Fw_*hrtAB* promoter | GGGGGAATTCCCAAAAAACTAAATTTGTAAAATCCGATTCACATTCCGTTTACAAAAC | Forward primer, *hrtAB* sequencing ladder (the same one used for the transcriptional fusion). |
| Rv_*hrtAB* promoter | CCCCGGATCCCGTAATTTTGCGTGTTTTAATTCTCTTAATGCC | Reverse primer, *hrtAB* sequencing ladder (the same one used for the transcriptional fusion). |
| NB probes | | |
| *lmo1634* NB probe | AGTGCCATTGCGTGAACGATATTGTCTACTTGTTCTTGGTTGTAACTTTCAAATGCTTTCAATGC | Single stranded probe for *lmo1634* mRNA |
| *hrtA* NB probe | GTTCCGCAATTACGAGTAACTGATCACGGAC | Single stranded probe for *hrtA* mRNA |
| *hrtB* NB probe | GAAAGCAGCTAGAACAAATGCGGCGATAACG | Single stranded probe for *hrtB* mRNA |
| *frvA* NB probe | ACTTTCTCCCGGCCGAACGAAGACCATATCATCCAGTTGTA | Single stranded probe for *frvA* mRNA |
| *fri* NB probe | ATGTAAAGTGAAGAAGTTGTGGCCTCTC | Single stranded probe for *fri* mRNA |
| *lmo1257* NB probe | TGGAGGAAGTCACCTGTCCACAGTTTTCCG | Single stranded probe for *lmo1257* mRNA |
| *recA* NB probe | TTGTTTTAAAGCTTGGTCTAATGCCGCTTG | Single stranded probe for *recA* mRNA |
| *ctaA* NB probe | CAACGACCGTCATACAAATAATCGTTAG | Single stranded probe for *ctaA* mRNA |
| *tcsA* ss NB probe | GTAAGCCTTCCCATGCTGATTGGTTAAACGAACGGTC | Single stranded probe for *tcsA* mRNA |
| *oppA* ss NB probe | GGAATGCGAATAGTGAGTTAATGTAAGCAGTTGG | Single stranded probe for *oppA* mRNA |
| *lmo0484* NB probe | CTTTTTCTACCTTAATCGTATTAGTTAC | Single stranded probe for *lmo0484* mRNA |
| *lmo2185* NB probe | GTTGTACCTGGATCAGGCTTTG | Single stranded probe for *lmo2185* mRNA |
| *lmo2186* NB probe | GTTAATCCAGTAGAAAGAAACGAAAAAC | Single stranded probe for *lmo2186* mRNA |
| 16S rRNA | GGCCATTACCCTACCAACTAGCTAATGCAC | Single stranded probe for 16S rRNA |
