# Supplementary material for: Listeria monocytogenes Relies on the Heme-Regulated Transporter hrtAB to Resist Heme Toxicity and Uses Heme as a Signal to Induce Transcription of lmo1634, Encoding Listeria Adhesion Protein
Source: Front Microbiol. 2018 Dec 18;9:3090. doi: 10.3389/fmicb.2018.03090 (PMC6305404; doi:10.3389/fmicb.2018.03090)
Supplement: Supplementary file 3 [file Table_3.DOCX]

Supplementary Table S3: Genes ≥ 4.0-fold up-regulated and ≤ - 4.0-fold down-regulated in *L. monocytogenes* wild-type cells stressed with 8 µM hemin for 30 min compared to non-stressed cells.

| Gene Symbol | Gene name | Regulation | Fold Change^[[1]](#endnote-1)^ | Product^b^ | COG^b^ |
| --- | --- | --- | --- | --- | --- |
| *lmo1634*^¥^ |  |  | 434,2 | Bifunctional acetaldehyde-CoA/alcohol dehydrogenase | Energy production and conversion |
| *lmo2580* | *hrtA* | HssRS^1^ | 276,3 | Heme efflux system ATPase HrtA | Defense/virulence mechanisms |
| *lmo2581* | *hrtB* | HssRS^1^ | 260,5 | Heme efflux system permease HrtB | Defense/virulence mechanisms |
| *lmo0641*^¥§^ | *frvA* | Fur^2^, PerR^3^ | 68,2 | Lead, cadmium, zinc and mercury transporting ATPase; Copper-translocating P-type ATPase | Inorganic ion transport and metabolism |
| *lmo1303* | *yneA* | LexA/RecA^4^ | 27,3 | Cell division suppressor protein YneA | Cell wall/membrane biogenesis |
| *lmo0102*^§^ |  |  | 22,0 | Antibiotic biosynthesis monooxygenase | Function unknown |
| *lmo0607* |  |  | 21,5 | Similar to ABC transporter, ATP-binding protein | Defense/virulence mechanisms |
| *lmo2271* |  | LexA/RecA^4^ | 21,2 | Hypothetical protein | Not in COGs |
| *lmo0606* |  |  | 21,1 | MarR family transcriptional regulator | Transcription |
| *lmo2828*^¤^ |  | LexA/RecA^4^ | 18,7 | Hypothetical protein | Not in COGs |
| *lmo0608* |  |  | 18,1 | Similar to ABC transporter, ATP-binding protein | Defense/virulence mechanisms |
| *lmo1257* |  |  | 17,7 | Hypothetical protein | Not in COGs |
| *lmo2785* | *kat* | Fur^2^, PerR^3^, SigB^5^ | 16,7 | Catalase | Inorganic ion transport and metabolism |
| *lmo0103*^§^ |  |  | 16,3 | NADH dehydrogenase | Energy production and conversion |
| *lmo2410*^¤^ |  |  | 15,6 | Hypothetical protein | Not in COGs |
| *lmo1975* | *dinB* | LexA/RecA^4^ | 14,5 | DNA polymerase IV | Replication, recombination and repair |
| *lmo2686* |  |  | 14,3 | Hypothetical protein | Not in COGs |
| *lmo1424* | *mntH* | SigB^5^ | 13,3 | Manganese transport protein MntH | Inorganic ion transport and metabolism |
| *lmo1421* |  | LexA/RecA^4^, SigB^5^ | 11,9 | Glycine betaine ABC transport system, ATP-binding protein | Defense/virulence mechanisms |
| *lmo1800* |  |  | 11,5 | Protein tyrosine phosphatase | Signal transduction mechanisms |
| *lmo1245*^¤^ |  |  | 11,3 | Hypothetical protein | Not in COGs |
| *lmo0943* | *fri* | Fur^2^, PerR^3^ | 10,1 | Non-heme iron-binding ferritin | Inorganic ion transport and metabolism |
| *lmo2432*^¤^ |  |  | 10,1 | Hypothetical protein | Not in COGs |
| *lmo2270* | *comK'* |  | 9,9 | Competence transcription factor | Transcription |
| *lmo0490* | *aroE* |  | 9,9 | Shikimate 5-dehydrogenase | Amino acid transport and metabolism |
| *lmo1690*^¤^ |  | LisR^6^ | 9,7 | Hypothetical protein | General function prediction only |
| *lmo2235* |  |  | 9,5 | Similar to NADH oxidase | Energy production and conversion;  General function prediction only |
| *lmo1849* | *sitB* | SigB^5^ | 9,1 | Manganese ABC transporter, ATP-binding protein SitB | Inorganic ion transport and metabolism |
| *lmo2269*^¤^ |  |  | 8,8 | Hypothetical protein | Not in COGs |
| *lmo2364* |  |  | 8,6 | Listeria RofA-like transcriptional regulator | Not in COGs |
| *lmo0496* |  | CtsR^7^ | 8,4 | Hypothetical protein | Function unknown |
| *lmo2210*^¤§^ |  | LisR^6^ | 8,4 | Hypothetical protein | Not in COGs |
| *lmo2238* |  |  | 8,1 | Major facilitator family transporter | Carbohydrate transport and metabolism;  Amino acid transport and metabolism; Inorganic ion transport and metabolism; General function prediction only |
| *lmo2489* | *uvrB* | LexA/RecA^4^ | 7,5 | Excinuclease ABC subunit B | Replication, recombination and repair |
| *lmo2237* |  |  | 7,4 | Transport protein | Carbohydrate transport and metabolism; Amino acid transport and metabolism; Inorganic ion transport and metabolism; General function prediction only |
| *lmo0912*^¥^ |  |  | 7,3 | Formate efflux transporter | Inorganic ion transport and metabolism |
| *lmo1422* |  | LexA/RecA^4^, SigB^5^ | 7,3 | Glycine betaine ABC transport system, permease protein | Defense/virulence mechanisms |
| *lmo2173* |  |  | 7,2 | Sigma-54 dependent transcriptional regulator | Transcription;  Signal transduction mechanisms |
| *lmo1516* |  | CodY^8^ | 7,0 | Ammonium transporter | Inorganic ion transport and metabolism |
| *lmo1848* | *sitD* | SigB^5^ | 7,0 | Manganese ABC transporter, inner membrane permease protein SitD | Inorganic ion transport and metabolism |
| *lmo1517* |  | CodY^8^ | 6,9 | Nitrogen regulatory protein P-II | Amino acid transport and metabolism |
| *lmo0200* | *prfA* | PrfA^9^ | 6,7 | Listeriolysin positive regulatory protein | Transcription |
| *lmo0976* |  |  | 6,6 | GNAT family acetyltransferase | Transcription;  General function prediction only |
| *lmo0411* |  |  | 6,5 | Phosphoenolpyruvate synthase | Carbohydrate transport and metabolism |
| *lmo2236* |  |  | 6,5 | Shikimate 5-dehydrogenase | Amino acid transport and metabolism |
| *lmo2187* |  |  | 6,5 | Hypothetical protein | Not in COGs |
| *lmo0589* |  |  | 6,3 | Hypothetical protein | Function unknown |
| *lmo0977* |  | CtsR^7^ | 6,2 | Putative acetyl esterase | Inorganic ion transport and metabolism |
| *lmo1921* |  |  | 6,2 | Hypothetical protein | Function unknown |
| *lmo2675* | *umuD* | LexA/RecA^4^ | 6,2 | Hypothetical protein | Not in COGs |
| *lmo0410* |  |  | 6,1 | Pseudo-gene | Not in COGs |
| *lmo0800* |  |  | 6,1 | Hypothetical protein | Function unknown |
| *lmo0613* |  |  | 6,0 | Bifunctional protein: zinc-containing alcohol dehydrogenase; quinone oxidoreductase (NADPH:quinone reductase); Similar to arginate lyase | Energy production and conversion;  General function prediction only |
| *lmo1252*^§^ |  |  | 5,8 | Membrane protein | Function unknown |
| *lmo0292* | *htrA* | LisR^6^ | 5,8 | Serine protease, DegP/HtrA, do-like | Posttranslational modification, protein turnover, chaperones |
| *lmo0450* |  |  | 5,8 | Hypothetical protein | Function unknown |
| *lmo2365* |  |  | 5,8 | Listeria RofA-like transcriptional regulator | Transcription;  Carbohydrate transport and metabolism |
| *lmo1714*^¤^ |  |  | 5,7 | Hypothetical protein | Not in COGs |
| *lmo1847* | *sitA* | SigB^5^ | 5,7 | Manganese ABC transporter, periplasmic-binding protein SitA | Cell wall/membrane biogenesis |
| *lmo1398* | *recA* | LexA/RecA^4^ | 5,7 | Recombinase A | Replication, recombination and repair |
| *lmo1102*^§^ |  |  | 5,6 | Cadmium efflux system accessory protein | Transcription |
| *lmo1413* |  |  | 5,6 | Peptidoglycan binding protein | Not in COGs |
| *llmo0997* | *clpE* | SigB^5^ | 5,6 | ATP-dependent protease | Posttranslational modification, protein turnover, chaperones |
| *lmo2709* |  |  | 5,5 | Hypothetical protein | Not in COGs |
| *lmo2460* |  |  | 5,5 | Transcriptional regulator, putative | Transcription |
| *lmo0881*^¤^ |  |  | 5,5 | Hypothetical protein | Not in COGs |
| *lmo1099* |  |  | 5,5 | Hypothetical protein | Transcription;  Signal transduction mechanisms |
| *lmo0518*^¤^ |  |  | 5,4 | Hypothetical protein | Function unknown |
| *lmo0232* | *clpC* | CtsR^7^ | 5,4 | Endopeptidase Clp ATP-binding chain C | Posttranslational modification, protein turnover, chaperones |
| *lmo2587*^¤^ |  |  | 5,4 | Hypothetical protein | Function unknown |
| *lmo0451* |  |  | 5,4 | Hypothetical protein | Signal transduction mechanisms |
| *lmo2488* | *uvrA* | LexA/RecA^4^ | 5,3 | Excinuclease ABC subunit A | Replication, recombination and repair |
| *lmo2170* |  |  | 5,3 | Enoyl-[acyl-carrier-protein] reductase | General function prediction only |
| *lmo1098* |  |  | 5,3 | Hypothetical protein | Not in COGs |
| *lmo0412* |  |  | 5,3 | Hypothetical protein | Not in COGs |
| *lmo0835* |  |  | 5,3 | Peptidoglycan binding protein | Not in COGs |
| *lmo0492* |  |  | 5,2 | Transcriptional regulator, LysR family | Transcription |
| *lmo2478* | *trxB* | PerR^3^ | 5,0 | Thioredoxin reductase | Posttranslational modification, protein turnover, chaperones |
| *lmo1518*^¤^ |  | LisR^6^ | 5,0 | Hypothetical protein | Not in COGs |
| *lmo2200* |  |  | 5,0 | Similar to transcription regulator | Transcription |
| *lmo1001*^¤^ |  |  | 4,8 | Hypothetical protein | Function unknown |
| *lmo2437*^¤^ |  |  | 4,6 | Hypothetical protein | General function prediction only |
| *lmo2199* |  |  | 4,6 | Organic hydroperoxide resistance protein | Posttranslational modification, protein turnover, chaperones |
| *lmo0902* |  |  | 4,6 | Transcriptional regulator, GntR family | Transcription |
| *lmo0788* |  | PrfA^9^ | 4,5 | Activator of (R)-2-hydroxyglutaryl-CoA dehydratase | Lipid transport and metabolism;  Function unknown |
| *lmo2492* |  |  | 4,4 | Hypothetical protein | Not in COGs |
| *lmo1919* |  | LisR^6^ | 4,4 | Probable metal-dependent peptidase | General function prediction only |
| *lmo0944* |  | CtsR^7^ | 4,4 | Hypothetical protein | Function unknown |
| *lmo0397*^¤^ |  |  | 4,4 | Hypothetical protein | Function unknown |
| *lmo0612*^§^ |  |  | 4,4 | Transcription regulator, MarR family | Transcription |
| *lmo2386* |  | SigB^5^ | 4,4 | Membrane protein | Function unknown |
| *lmo1220* |  |  | 4,4 | Transcriptional regulator, HxlR family | Transcription |
| *lmo1056* |  |  | 4,3 | Hypothetical protein | Not in COGs |
| *lmo0836* | *psiE* |  | 4,3 | Phosphate-starvation-inducible protein PsiE | Function unknown |
| *lmo2827*^§^ |  | CtsR^7^ | 4,3 | Transcriptional regulator, MarR family | Transcription |
| *lmo0355*^¥^ |  |  | 4,2 | Fumarate reductase flavoprotein subunit | Energy production and conversion |
| *lmo1618*^§^ |  |  | 4,2 | Transcriptional regulator, MarR family | Transcription |
| *lmo0592* |  |  | 4,2 | Hypothetical protein | Not in COGs |
| *lmo0822* |  | CtsR^7^ | 4,2 | Transcriptional regulator, MerR family | Transcription |
| *lmo2568*^¤^ |  |  | 4,1 | Hypothetical protein | Not in COGs |
| *lmo0160* |  |  | 4,1 | Peptidoglycan binding protein | Cell wall/membrane biogenesis |
| *lmo2459* | *gap* |  | 4,1 | Glyceraldehyde-3-phosphate dehydrogenase | Carbohydrate transport and metabolism |
| *lmo1956* | *fur* | Fur^2^, PerR^3^ | 4,1 | Ferric uptake regulation protein FUR | Inorganic ion transport and metabolism |
| *lmo0871* |  |  | 4,1 | Transcriptional regulator, HxlR family | Transcription |
| *lmo1447* | *zurA* |  | 4,1 | Zinc transport protein (ABC transporter, ATP-binding protein) | Inorganic ion transport and metabolism |
| *lmo0964* |  |  | 4,1 | GTP pyrophosphokinase domain | Secondary metabolites biosynthesis, transport and catabolism |
| *lmo0588* |  |  | 4,0 | Deoxyribodipyrimidine photolyase | Replication, recombination and repair |
| *lmo0747* |  |  | 4,0 | Hypothetical protein | Not in COGs |
| *lmo2363* |  |  | 4,0 | Glutamate decarboxylase | Amino acid transport and metabolism |
| *lmo0832* | *orfA* |  | 4,0 | Transposase OrfA, IS3 family | Replication, recombination and repair |
| *lmo2186*^§^ | *hbp1/ isdC* | Fur^2^ | -369,3 | NPQTN cell wall anchored protein IsdC | Cell wall/membrane biogenesis |
| *lmo1999*^¥§^ |  | CodY^8^ | -226,2 | Glucosamine--fructose-6-phosphate aminotransferase | Cell wall/membrane biogenesis |
| *lmo2185*^§^ | *hbp2/ isdA* | Fur^2^ | -190,6 | Cell surface protein IsdA, transfers heme from hemoglobin to apo-IsdC | Cell wall/membrane biogenesis |
| *lmo2002*^§^ |  |  | -170,3 | PTS system, mannose-specific IIB component | Carbohydrate transport and metabolism |
| *lmo0541*^§^ |  | Fur^2^ | -167,9 | ABC transporter substrate-binding protein | Inorganic ion transport and metabolism |
| *lmo2184*^§^ | *isdE* | Fur^2^ | -143,3 | Heme transporter IsdDEF, lipoprotein IsdE | Inorganic ion transport and metabolism |
| *lmo2003*^§^ |  |  | -136,3 | Transcriptional regulator, GntR family | Transcription |
| *lmo2650*^¥§^ |  |  | -118,1 | PTS system, lactose/cellobiose specific IIB subunit | Carbohydrate transport and metabolism |
| *lmo0362* | *tatA* | CtsR^7^ | -117,9 | Twin-arginine translocation protein TatA | Intracellular trafficking and secretion |
| *lmo2181* | *srtB* | Fur^2^ | -115,6 | NPQTN specific sortase B | Cell wall/membrane biogenesis |
| *lmo2651*^¥§^ |  |  | -112,8 | PTS system, IIA component | Carbohydrate transport and metabolism; Signal transduction mechanisms |
| *lmo2183*^§^ | *isdF* | Fur^2^ | -110,5 | Heme transporter IsdDEF, permease component IsdF | Inorganic ion transport and metabolism |
| *lmo2000*^§^ |  |  | -105,7 | PTS system, mannose-specific IID component | Carbohydrate transport and metabolism |
| *lmo2004*^§^ |  |  | -100,4 | Transcriptional regulator, GntR family | Transcription |
| *lmo2648*^¥§^ |  |  | -95,7 | Phosphotriesterase family protein | General function prediction only |
| *lmo0365* | *efeU* | Fur^2^ | -86,1 | Ferrous iron transport permease EfeU | Inorganic ion transport and metabolism |
| *lmo2649*^¥§^ |  |  | -82,4 | PTS system ascorbate-specific transporter subunit IIC | Function unknown |
| *lmo2647*^¥§^ |  | CodY^8^ | -82,0 | Creatinine amidohydrolase | General function prediction only |
| *lmo0517*^§^ |  |  | -77,0 | Hypothetical phosphatase | Carbohydrate transport and metabolism |
| *lmo2180* |  | Fur^2^ | -74,2 | Hypothetical protein | Not in COGs |
| *lmo0366* | *efeO* | Fur^2^ | -70,5 | Ferrous iron transport periplasmic protein EfeO | Inorganic ion transport and metabolism |
| *lmo1007*^§^ |  | Fur^2^, CtsR^7^ | -63,8 | Hypothetical protein | Not in COGs |
| *lmo2708*^¥§^ |  |  | -63,2 | PTS system, cellobiose-specific IIC component | Carbohydrate transport and metabolism |
| *lmo2182* |  | Fur^2^ | -61,4 | Heme transporter analogous to IsdDEF, ATP-binding protein | Inorganic ion transport and metabolism; Coenzyme transport and metabolism |
| *lmo0023* |  |  | -58,6 | PTS system, mannose-specific IIC component | Carbohydrate transport and metabolism |
| *lmo0915*^¥§^ |  |  | -56,6 | PTS sugar transporter subunit IIC | Carbohydrate transport and metabolism |
| *lmo0361* | *tatC* | CtsR^7^ | -54,6 | Sec-independent protein secretion pathway component TatC | Intracellular trafficking and secretion |
| *lmo0027*^¥§^ |  |  | -54,2 | PTS beta-glucoside transporter subunit IIABC | Carbohydrate transport and metabolism |
| *lmo0916*^¥§^ |  |  | -51,9 | PTS sugar transporter subunit IIA | Carbohydrate transport and metabolism |
| *lmo1998*^¥§^ |  | CodY^8^ | -50,2 | Glucosamine--fructose-6-phosphate aminotransferase | Cell wall/membrane biogenesis |
| *lmo2125*^§^ | *malE* |  | -45,4 | Maltose/maltodextrin ABC transporter, substrate binding periplasmic protein MalE | Carbohydrate transport and metabolism |
| *lmo2123* | *malG* |  | -43,8 | Maltose/maltodextrin ABC transporter, permease protein MalG | Carbohydrate transport and metabolism |
| *lmo1054*^¥§^ | *pdhC* |  | -43,1 | Dihydrolipoamide acetyltransferase | Energy production and conversion |
| *lmo1053*^¥§^ | *pdhB* |  | -43,0 | Pyruvate dehydrogenase E1 component beta subunit | Energy production and conversion |
| *lmo1052*^¥§^ | *pdhA* |  | -41,2 | Pyruvate dehydrogenase E1 component alpha subunit | Energy production and conversion |
| *lmo0024* |  |  | -40,3 | PTS system, mannose-specific IID component | Carbohydrate transport and metabolism |
| *lmo0914*^¥§^ |  |  | -38,9 | PTS sugar transporter subunit IIB | Carbohydrate transport and metabolism |
| *lmo2646*^¥§^ |  | CodY^8^ | -37,6 | Hypothetical protein | Not in COGs |
| *lmo2124*^¥^ | *malF* |  | -36,2 | Maltose/maltodextrin ABC transporter, permease protein MalF | Carbohydrate transport and metabolism |
| *lmo2122* | *malA* |  | -34,1 | Maltodextrose utilization protein MalA | Function unknown |
| *lmo0400*^§^ |  |  | -33,9 | PTS system, fructose-specific, IIC component | Carbohydrate transport and metabolism |
| *lmo1997*^§^ |  | CodY^8^ | -33,8 | PTS mannose transporter subunit IIA | Carbohydrate transport and metabolism |
| *lmo0180*^§^ |  |  | -33,5 | N-Acetyl-D-glucosamine ABC transport system, permease protein 2 | Carbohydrate transport and metabolism |
| *lmo0347* | *dhaL* |  | -30,3 | Phosphoenolpyruvate-dihydroxyacetone phosphotransferase, ADP-binding subunit DhaL | Carbohydrate transport and metabolism |
| *lmo0351* |  |  | -28,8 | Phosphotransferase mannnose-specific family component IIA | Function unknown |
| *lmo0484*^§^ | *isdG* | Fur^2^ | -28,3 | Heme-degrading monooxygenase IsdG | General function prediction only |
| *lmo0847* | *glnP* |  | -27,9 | Glutamine transport system permease protein GlnP | Amino acid transport and metabolism; Signal transduction mechanisms |
| *lmo2799*^¥§^ |  |  | -27,5 | PTS system, mannitol-specific IIB component/ PTS system, mannitol-specific IIC component | Carbohydrate transport and metabolism |
| *lmo0135* |  |  | -27,5 | Oligopeptide ABC transporter, periplasmic oligopeptide-binding protein | Amino acid transport and metabolism |
| *lmo1348*^¥^ | *gcvT* |  | -27,4 | Glycine cleavage system aminomethyltransferase T | Amino acid transport and metabolism |
| *lmo2001*^§^ |  | CodY^8^ | -26,7 | PTS system, mannose-specific IIC component | Carbohydrate transport and metabolism |
| *lmo0137* |  |  | -26,0 | Oligopeptide transport system permease protein | Amino acid transport and metabolism; Inorganic ion transport and metabolism |
| *lmo0130*^¥§^ |  |  | -26,0 | 5'-nucleotidase | Nucleotide transport and metabolism |
| *lmo1389* |  |  | -26,0 | Unspecified monosaccharide ABC transport system, ATP-binding protein | General function prediction only |
| *lmo0348* | *dhaK* |  | -25,9 | Phosphoenolpyruvate-dihydroxyacetone phosphotransferase, dihydroxyacetone binding subunit DhaK | Carbohydrate transport and metabolism |
| *lmo0350* |  |  | -25,1 | Hypothetical protein | Not in COGs |
| *lmo0136* |  |  | -24,7 | Oligopeptide transport system permease protein | Amino acid transport and metabolism; Inorganic ion transport and metabolism |
| *lmo0278*^¥§^ |  |  | -23,4 | Multiple sugar ABC transporter, ATP-binding protein | Carbohydrate transport and metabolism |
| *lmo1055*^¥§^ | *pdhD* | SigB^5^ | -22,9 | Dihydrolipoamide dehydrogenase | Energy production and conversion |
| *lmo1958*^§^ | *fhuB* | Fur^2^ | -22,5 | ABC-type Fe3+-siderophore transport system, permease component | Inorganic ion transport and metabolism |
| *lmo1390*^¥^ |  |  | -22,0 | Unspecified monosaccharide ABC transport system, permease component | General function prediction only |
| *lmo2616* | *rplR* |  | -20,3 | 50S ribosomal protein L18 | Translation |
| *lmo0848* |  |  | -20,2 | Glutamate transport ATP-binding protein | Amino acid transport and metabolism |
| *lmo2090*^¥^ | *argG* | CodY^8^ | -20,1 | Argininosuccinate synthase | Amino acid transport and metabolism |
| *lmo2163*^¥§^ |  |  | -19,9 | Myo-inositol 2-dehydrogenase 1 | General function prediction only |
| *lmo0903* |  |  | -19,9 | OsmC/Ohr family protein | Posttranslational modification, protein turnover, chaperones |
| *lmo1957*^§^ | *fhuG* | Fur^2^ | -19,6 | ABC-type Fe3+-siderophore transport system, permease 2 component | Inorganic ion transport and metabolism |
| *lmo1349*^¥^ |  |  | -19,1 | Glycine dehydrogenase subunit 1 | Amino acid transport and metabolism |
| *lmo0181*^§^ |  |  | -18,9 | N-Acetyl-D-glucosamine ABC transport system, sugar-binding protein | Carbohydrate transport and metabolism |
| *lmo2617* | *rplF* |  | -18,7 | 50S ribosomal protein L6 | Translation |
| *lmo2618* | *rpsH* | SigB^5^ | -18,6 | 30S ribosomal protein S8 | Translation |
| *lmo1882* | *rpsN* |  | -18,1 | 30S ribosomal protein S14 | Translation |
| *lmo2621* | *rplX* |  | -18,0 | 50S ribosomal protein L24 | Translation |
| *lmo2162*^¥§^ |  |  | -17,7 | Inosose dehydratase | Carbohydrate transport and metabolism |
| *lmo2615* | *rpsE* |  | -17,5 | 30S ribosomal protein S5 | Translation |
| *lmo2620* | *rplE* |  | -17,1 | 50S ribosomal protein L5 | Translation |
| *lmo2622* | *rplN* | SigB^5^ | -17,0 | 50S ribosomal protein L14 | Translation |
| *lmo0367*^§^ | *efeB* | Fur^2^ | -16,0 | Ferrous iron transport peroxidase EfeB | Inorganic ion transport and metabolism |
| *lmo0105* |  |  | -15,4 | Chitinase | Carbohydrate transport and metabolism; General function prediction only |
| *lmo1391*^¥^ |  |  | -15,2 | Unspecified monosaccharide ABC transport system, permease component 2 | General function prediction only |
| *lmo2623* | *rpsQ* |  | -15,1 | 30S ribosomal protein S17 | Translation |
| *lmo2614* | *rpmD* |  | -15,1 | 50S ribosomal protein L30 | Translation |
| *lmo2800*^¥§^ |  |  | -14,9 | Oxidoreductase, Gfo/Idh/MocA family | General function prediction only |
| *lmo0813*^§^ |  | CodY^8^ | -14,8 | Fructokinase | Transcription;  Carbohydrate transport and metabolism |
| *lmo0179*^§^ |  |  | -13,8 | N-Acetyl-D-glucosamine ABC transport system, permease protein 1 | Carbohydrate transport and metabolism |
| *lmo2624* | *rpmC* |  | -13,5 | 50S ribosomal protein L29 | Translation |
| *lmo0301* |  |  | -13,3 | PTS system, cellobiose-specific IIA component | Carbohydrate transport and metabolism |
| *lmo0152* |  |  | -13,3 | Oligopeptide ABC transporter, periplasmic oligopeptide-binding protein | Amino acid transport and metabolism |
| *lmo2625* | *rplP* |  | -13,2 | 50S ribosomal protein L16 | Translation |
| *lmo1971* | *ulaA* |  | -13,1 | PTS system ascorbate-specific transporter subunit IIC | Function unknown |
| *lmo2772* |  |  | -13,0 | PTS system, beta-glucoside-specific IIB component/ PTS system, beta-glucoside-specific IIC component/ PTS system, beta-glucoside-specific IIA component | Carbohydrate transport and metabolism |
| *lmo0861* |  |  | -13,0 | Sugar ABC transporter, permease protein precursor | Carbohydrate transport and metabolism |
| *lmo2126* |  |  | -12,9 | Neopullulanase | Carbohydrate transport and metabolism |
| *lmo0863* |  |  | -12,8 | Hypothetical protein | Not in COGs |
| *lmo0766* |  |  | -12,7 | Sugar ABC transporter permease, putative | Carbohydrate transport and metabolism |
| *lmo0545* |  |  | -12,2 | Glucitol operon activator protein | Transcription |
| *lmo2626* | *rpsC* | Sig^B^ | -12,1 | 30S ribosomal protein S3 | Translation |
| *lmo1960*^§^ | *fhuC* | Fur^2^, CtsR^7^ | -12,1 | ABC-type Fe3+-siderophore transport system, ATPase component | Inorganic ion transport and metabolism; Coenzyme transport and metabolism |
| *lmo0767* |  |  | -12,0 | ABC transporter, permease protein | Carbohydrate transport and metabolism |
| *lmo0346* |  |  | -11,9 | Triosephosphate isomerase | Carbohydrate transport and metabolism |
| *lmo0859* |  |  | -11,8 | Multiple sugar ABC transporter, substrate-binding protein | Carbohydrate transport and metabolism |
| *lmo1131* | *cydC* | Fur^2^ | -11,7 | Transport ATP-binding protein CydC | Energy production and conversion; Posttranslational modification, protein turnover, chaperones; |
| *lmo2161*^¥§^ |  |  | -11,5 | Hypothetical protein | Carbohydrate transport and metabolism |
| *lmo1731* |  |  | -11,4 | N-Acetyl-D-glucosamine ABC transport system, permease protein 1 | Carbohydrate transport and metabolism |
| *lmo0862* |  |  | -11,3 | Trehalose-6-phosphate hydrolase | Carbohydrate transport and metabolism |
| *lmo1641* | *citB* |  | -11,2 | Aconitate hydratase | Energy production and conversion |
| *lmo1959* | *fhuD* | Fur^2^ | -11,2 | Ferrichrome-binding periplasmic protein precursor | Inorganic ion transport and metabolism |
| *lmo0735* |  | SigB^5^ | -11,1 | D-allulose-6-phosphate 3-epimerase | Carbohydrate transport and metabolism |
| *lmo2627* | *rplV* | SigB^5^ | -10,8 | 50S ribosomal protein L22 | Translation |
| *lmo0973* | *dltB* | VirRS^10^ | -10,6 | DltB protein for D-alanine esterification of lipoteichoic acid and wall teichoic acid | Cell wall/membrane biogenesis |
| *lmo0178*^§^ |  |  | -10,6 | Putative ROK-family transcriptional regulator | Transcription;  Carbohydrate transport and metabolism |
| *lmo2115* | *bceB* | VirRS^10^ | -10,5 | Bacitracin export permease protein BceB | Defense/virulence mechanisms |
| *lmo2121* |  |  | -10,4 | Maltose phosphorylase | Carbohydrate transport and metabolism |
| *lmo1973* |  |  | -10,4 | Hypothetical protein | Carbohydrate transport and metabolism; Signal transduction mechanisms |
| *lmo2409* |  |  | -10,3 | Hypothetical protein | Not in COGs |
| *lmo0679* | *flhB* |  | -10,2 | Flagellar biosynthesis protein FlhB | Cell motility;  Intracellular trafficking and secretion |
| *lmo2105*^§^ | *feoB* | Fur^2^ | -10,2 | Ferrous iron transport protein B | Inorganic ion transport and metabolism |
| *lmo2851*^§^ |  |  | -10,2 | Transcriptional regulator of rhamnose utilization, AraC family | Transcription |
| *lmo0972* | *dltC* | VirRS^10^ | -10,1 | D-alanine--poly(phosphoribitol) ligase subunit 2 | Cell wall/membrane biogenesis |
| *lmo2771* |  |  | -10,0 | 6-phospho-beta-glucosidase | Carbohydrate transport and metabolism |
| *lmo1388* | *tcsA* |  | -9,9 | CD4+ T cell-stimulating antigen, lipoprotein | General function prediction only |
| *lmo2628* | *rpsS* | SigB^5^ | -9,9 | 30S ribosomal protein S19 | Translation |
| *lmo2611* | *adk* |  | -9,7 | Adenylate kinase | Nucleotide transport and metabolism |
| *lmo2801*^§^ |  |  | -9,7 | N-acetylmannosamine-6-phosphate 2-epimerase | Carbohydrate transport and metabolism |
| *lmo2114* | *bceA* | CtsR^7^, VirRS^10^ | -9,5 | Bacitracin export ATP-binding protein BceA | Defense/virulence mechanisms |
| *lmo2665*^¥^ |  |  | -9,5 | PTS system, galactitol-specific IIC component | Carbohydrate transport and metabolism |
| *lmo2325* |  |  | -9,3 | Hypothetical protein | Not in COGs |
| *lmo2160*^¥§^ |  |  | -9,3 | Inosose isomerase | Carbohydrate transport and metabolism |
| *lmo1832* | *pyrF* |  | -9,2 | Orotidine 5'-phosphate decarboxylase | Nucleotide transport and metabolism |
| *lmo2135* |  |  | -9,1 | PTS system, fructose-specific IIBC component | Carbohydrate transport and metabolism |
| *lmo1961*^§^ |  | Fur^2^ | -9,1 | Thioredoxin reductase | Posttranslational modification, protein turnover, chaperones |
| *lmo0917*^¥§^ |  |  | -9,0 | 6-phospho-beta-glucosidase | Carbohydrate transport and metabolism |
| *lmo1730* |  |  | -9,0 | N-Acetyl-D-glucosamine ABC transport system, sugar-binding protein | Carbohydrate transport and metabolism |
| *lmo0971* | *dltD* | VirRS^10^ | -9,0 | DltD protein for D-alanine esterification of lipoteichoic acid and wall teichoic acid | Cell wall/membrane biogenesis |
| *lmo0032* |  |  | -8,9 | Sugar kinase and transcription regulator | Transcription;  Carbohydrate transport and metabolism |
| *lmo2250* | *arpJ* |  | -8,8 | Amino acid ABC transporter, amino acid-binding/permease protein | Amino acid transport and metabolism; Signal transduction mechanisms |
| *lmo2773*^§^ |  |  | -8,8 | Beta-glucoside bgl operon antiterminator, BglG family | Transcription |
| *lmo0876* |  |  | -8,7 | Similar to PTS system, Lichenan-specific enzyme IIC component | Carbohydrate transport and metabolism |
| *lmo2104* |  | Fur^2^ | -8,7 | Hypothetical protein | Inorganic ion transport and metabolism |
| *lmo2101* | *pdxS* |  | -8,7 | Pyridoxal biosynthesis lyase PdxS | Coenzyme transport and metabolism |
| *lmo1831* | *pyrE* | SigB^5^ | -8,5 | Orotate phosphoribosyltransferase | Nucleotide transport and metabolism |
| *lmo1261* |  | SigB^5^ | -8,4 | Membrane protein | Function unknown |
| *lmo2683* |  |  | -8,3 | PTS system, cellobiose-specific IIB component | Carbohydrate transport and metabolism |
| *lmo2629* | *rplB* | SigB^5^ | -8,2 | 50S ribosomal protein L2 | Translation |
| *lmo0697* | *flgE* |  | -8,1 | Flagellar hook protein FlgE | Cell motility |
| *lmo2630* | *rplW* |  | -8,1 | 50S ribosomal protein L23 | Translation |
| *lmo1766* | *purN* |  | -8,1 | Phosphoribosylglycinamide formyltransferase | Nucleotide transport and metabolism |
| *lmo1729*^¥^ |  |  | -8,0 | Beta-glucosidase | Carbohydrate transport and metabolism |
| *lmo0182*^§^ |  |  | -7,9 | Glycosyl hydrolase, family 31 | Carbohydrate transport and metabolism |
| *lmo1132* | *cydD* | Fur^2^ | -7,9 | Transport ATP-binding protein CydD | Defense/virulence mechanisms |
| *lmo0643* |  |  | -7,9 | Transaldolase | Carbohydrate transport and metabolism |
| *lmo0731* |  |  | -7,9 | Hypothetical protein | Not in COGs |
| *lmo0014*^¥^ | *qoxB* | CtsR^7^ | -7,8 | AA3-600 quinol oxidase subunit I | Energy production and conversion |
| *lmo0300*^¥^ |  |  | -7,6 | Beta-glucosidase | Carbohydrate transport and metabolism |
| *lmo0015*^¥^ | *qoxC* |  | -7,6 | AA3-600 quinol oxidase subunit III | Energy production and conversion |
| *lmo0097* | *mptC* |  | -7,6 | PTS system, mannose-specific IIC component | Carbohydrate transport and metabolism |
| *lmo1879*^¥§^ | *cspD* |  | -7,6 | Cold shock protein | Transcription |
| *lmo0013*^¥^ | *qoxA* |  | -7,5 | AA3-600 quinol oxidase subunit II | Energy production and conversion |
| *lmo2102* | *pdxT* |  | -7,5 | Glutamine amidotransferase subunit PdxT | Coenzyme transport and metabolism |
| *lmo0860* | *msmF* |  | -7,5 | Multiple sugar ABC transporter, membrane-spanning permease protein MsmF | Carbohydrate transport and metabolism |
| *lmo2668* |  |  | -7,5 | Predicted galactitol operon regulator (Transcriptional antiterminator), BglG family / PTS system, mannitol/fructose-specific IIA component | Transcription;  Carbohydrate transport and metabolism; Signal transduction mechanisms |
| *lmo2251* |  |  | -7,4 | Amino acid ABC transporter, ATP-binding protein | Amino acid transport and metabolism |
| *lmo2850* | *rhaY* |  | -7,4 | Predicted L-rhamnose permease RhaY | Carbohydrate transport and metabolism; Amino acid transport and metabolism; Inorganic ion transport and metabolism; General function prediction only |
| *lmo2612* | *secY* |  | -7,4 | Preprotein translocase subunit SecY | Intracellular trafficking and secretion |
| *lmo1350*^¥^ |  |  | -7,4 | Glycine dehydrogenase subunit 2 | Amino acid transport and metabolism |
| *lmo0703* |  |  | -7,4 | UDP-N-acetylenolpyruvoylglucosamine reductase | Not in COGs |
| *lmo0765* |  |  | -7,4 | Hypothetical protein | Function unknown |
| *lmo2631* | *rplD* | SigB^5^ | -7,3 | 50S ribosomal protein L4 | Translation |
| *lmo2840* |  |  | -7,3 | Putative sucrose phosphorylase | Carbohydrate transport and metabolism |
| *lmo0016*^¥§^ | *qoxD* |  | -7,3 | AA3-600 quinol oxidase subunit IV | Energy production and conversion |
| *lmo1043*^¥^ | *mobB* |  | -7,2 | Molybdopterin-guanine dinucleotide biosynthesis protein MobB | Coenzyme transport and metabolism |
| *lmo1732* |  |  | -7,2 | N-Acetyl-D-glucosamine ABC transport system, permease protein 2 | Carbohydrate transport and metabolism |
| *lmo2664* |  | CodY^8^ | -7,2 | Galactitol-1-phosphate 5-dehydrogenase | Amino acid transport and metabolism; General function prediction only |
| *lmo1044*^¥^ | *moaE* |  | -7,2 | Molybdenum cofactor biosynthesis protein MoaE | Coenzyme transport and metabolism |
| *lmo0736* |  | SigB^5^ | -7,1 | Ribose-5-phosphate isomerase B | Carbohydrate transport and metabolism |
| *lmo0806* |  |  | -7,0 | Transcriptional regulator, MerR family, near polyamine transporter | Transcription |
| *lmo0096* | *mptA* |  | -7,0 | PTS system, mannose-specific IIB component/ PTS system, mannose-specific IIA component | Carbohydrate transport and metabolism |
| *lmo2341*^§^ |  |  | -7,0 | Pseudouridine kinase | Transcription;  Carbohydrate transport and metabolism |
| *lmo1867*^§^ |  |  | -7,0 | Pyruvate phosphate dikinase | Carbohydrate transport and metabolism |
| *lmo2613* | *rplO* |  | -6,9 | 50S ribosomal protein L15 | Translation |
| *lmo0778* |  |  | -6,8 | Hypothetical protein | Not in COGs |
| *lmo2433* |  | CtsR^7^ | -6,8 | Putative esterase | General function prediction only |
| *lmo2584*^¥^ |  |  | -6,7 | Formate dehydrogenase accessory protein | Energy production and conversion |
| *lmo0345* |  | CodY^8^ | -6,6 | Ribose 5-phosphate isomerase B | Carbohydrate transport and metabolism |
| *lmo1551* | *folC* |  | -6,6 | Dihydrofolate synthase/ Folylpolyglutamate synthase | Coenzyme transport and metabolism |
| *lmo0768* |  | CodY^8^ | -6,6 | Sugar ABC transporter, sugar-binding protein | Carbohydrate transport and metabolism |
| *lmo2831*^¥^ |  |  | -6,6 | Beta-phosphoglucomutase | General function prediction only |
| *lmo0782*^¥^ |  | SigB^5^ | -6,6 | PTS system, mannose-specific IIC component/ PTS system, fructose-specific IIC component | Carbohydrate transport and metabolism |
| *lmo1833* | *pyrD* |  | -6,4 | Dihydroorotate dehydrogenase 1B | Nucleotide transport and metabolism |
| *lmo0974* | *dltA* | VirRS^10^ | -6,4 | D-alanine--poly(phosphoribitol) ligase subunit 1 | Cell wall/membrane biogenesis |
| *lmo0810* | *potD* |  | -6,4 | ABC transporter, periplasmic spermidine putrescine-binding protein PotD | Amino acid transport and metabolism |
| *lmo1045*^¥^ | *moaD* |  | -6,4 | Molybdenum cofactor biosynthesis protein MoaD | Coenzyme transport and metabolism |
| *lmo2099* |  |  | -6,3 | Predicted galactitol operon regulator (Transcriptional antiterminator), BglG family / PTS system, mannitol/fructose-specific IIA component | Transcription;  Carbohydrate transport and metabolism; Signal transduction mechanisms |
| *lmo0298* |  | CodY^8^ | -6,3 | PTS system, cellobiose-specific IIC component | Carbohydrate transport and metabolism |
| *lmo0707* | *fliD* | CodY^8^ | -6,3 | Flagellar capping protein | Cell motility |
| *lmo0250* | *rplJ* |  | -6,3 | 50S ribosomal protein L10 | Translation |
| *lmo0808* | *potB* |  | -6,2 | Spermidine Putrescine ABC transporter permease component PotB | Amino acid transport and metabolism |
| *lmo0343* |  |  | -6,2 | Putative translaldolase | Carbohydrate transport and metabolism |
| *lmo0299*^¥^ |  | CodY^8^ | -6,2 | PTS system, cellobiose-specific IIB component | Carbohydrate transport and metabolism |
| *lmo0783*^¥^ |  | SigB^5^ | -6,1 | PTS system, mannose-specific IIB component | Carbohydrate transport and metabolism |
| *lmo0784*^¥^ |  | SigB^5^ | -6,1 | PTS system, mannose-specific IIB component/ PTS system, mannose-specific IIA component | Carbohydrate transport and metabolism |
| *lmo0021* |  |  | -6,1 | PTS system, IIA component | Carbohydrate transport and metabolism |
| *lmo2159*^¥§^ |  |  | -6,1 | Myo-inositol 2-dehydrogenase | General function prediction only |
| *lmo2607* | *rpsK* |  | -6,1 | 30S ribosomal protein S11 | Translation |
| *lmo2632* | *rplC* |  | -6,1 | 50S ribosomal protein L3 | Translation |
| *lmo2609* | *rpmJ* |  | -6,0 | 50S ribosomal protein L36 | Translation |
| *lmo0251* | *rplL* |  | -6,0 | 50S ribosomal protein L7/L12 | Translation |
| *lmo2608* | *rpsM* |  | -6,0 | 30S ribosomal protein S13 | Translation |
| *lmo2129* |  |  | -6,0 | Hypothetical protein | Not in COGs |
| *lmo1666* |  |  | -6,0 | Peptidoglycan linked protein (LPXTG) | Not in COGs |
| *lmo0809* | *potC* |  | -6,0 | Spermidine Putrescine ABC transporter permease component potC | Amino acid transport and metabolism |
| *lmo2586*^¥§^ |  |  | -6,0 | Formate dehydrogenase related protein | General function prediction only |
| *lmo1834* | *pyrDII* |  | -5,9 | Dihydroorotate dehydrogenase electron transfer subunit | Coenzyme transport and metabolism; Energy production and conversion |
| *lmo1653* |  |  | -5,8 | Putative cellsurface protein | Not in COGs |
| *lmo0599* |  |  | -5,8 | Transcriptional regulator, PadR family | Transcription |
| *lmo0706* | *flgL* |  | -5,8 | Flagellar hook-associated protein FlgL | Cell motility |
| *lmo0050* | *comD* |  | -5,7 | Histidine kinase of the competence regulon ComD | Signal transduction mechanisms |
| *lmo0384* |  |  | -5,7 | 5-deoxy-glucuronate isomerase | Carbohydrate transport and metabolism |
| *lmo2430* |  | Fur^2^ | -5,7 | ABC-type Fe3+-siderophore transport system, permease component | Inorganic ion transport and metabolism |
| *lmo1993*^§^ | *pdp* |  | -5,6 | Pyrimidine-nucleoside phosphorylase | Nucleotide transport and metabolism |
| *lmo0344* |  | CodY^8^ | -5,6 | Short chain dehydrogenase | Lipid transport and metabolism; Secondary metabolites biosynthesis, transport and catabolism;  General function prediction only |
| *lmo2654* | *fus* |  | -5,6 | Elongation factor G | Translation |
| *lmo1538*^¥§^ | *glpK* |  | -5,6 | Glycerol kinase | Energy production and conversion |
| *lmo0049* | *agrD* |  | -5,5 | Accessory gene regulator protein D, putative | Not in COGs |
| *lmo2849* |  |  | -5,5 | Rhamnulokinase | Carbohydrate transport and metabolism |
| *lmo0877* |  |  | -5,5 | Glucosamine-6-phosphate deaminase | Carbohydrate transport and metabolism |
| *lmo0051* | *comE* |  | -5,5 | Response regulator of the competence regulon ComE | Transcription;  Signal transduction mechanisms |
| *lmo2569*^¥§^ |  |  | -5,5 | Oligopeptide ABC transporter, periplasmic oligopeptide-binding protein | Amino acid transport and metabolism |
| *lmo2610* | *infA* |  | -5,5 | Translation initiation factor IF-1 | Translation |
| *lmo2606* | *rpoA* |  | -5,5 | DNA-directed RNA polymerase subunit alpha | Transcription |
| *lmo2156* |  | VirRS^10^ | -5,5 | Hypothetical protein | Function unknown |
| *lmo0546* |  |  | -5,5 | Sorbitol-6-phosphate 2-dehydrogenase | Amino acid transport and metabolism |
| *lmo2252* |  |  | -5,4 | Aspartate aminotransferase | Amino acid transport and metabolism |
| *lmo0017* | *capA* |  | -5,4 | Capsule biosynthesis protein capA | Cell wall/membrane biogenesis |
| *lmo0025* |  |  | -5,3 | Similar to phosphoheptose isomerase | General function prediction only |
| *lmo1191* | *cbiA* |  | -5,3 | Cobyrinic acid a,c-diamide synthase | Coenzyme transport and metabolism |
| *lmo2585*^¥§^ |  | CodY^8^ | -5,3 | Hypothetical protein | Function unknown |
| *lmo0183*^§^ |  | CodY^8^ | -5,3 | Alpha-glucosidase | Carbohydrate transport and metabolism |
| *lmo1042*^¥^ | *moeA* |  | -5,2 | Molybdopterin biosynthesis protein MoeA | Coenzyme transport and metabolism |
| *lmo0781*^¥^ |  | SigB^5^ | -5,2 | PTS system mannose-specific transporter subunit IID | Carbohydrate transport and metabolism |
| *lmo2684* |  |  | -5,2 | PTS system, cellobiose-specific IIC component | Carbohydrate transport and metabolism |
| *lmo0700* |  |  | -5,2 | Flagellar motor switch protein | Cell motility;  Intracellular trafficking and secretion; Signal transduction mechanisms |
| *lmo1783* | *rplT* |  | -5,1 | 50S ribosomal protein L20 | Translation |
| *lmo1255*^¥§^ |  |  | -5,1 | PTS system, trehalose-specific IIB component/ PTS system, trehalose-specific IIC component | Carbohydrate transport and metabolism |
| *lmo2605* | *rplQ* |  | -5,1 | 50S ribosomal protein L17 | Translation |
| *lmo1046*^¥^ | *moaC* |  | -5,0 | Molybdenum cofactor biosynthesis protein MoaC | Coenzyme transport and metabolism |
| *lmo1785* | *infC* | SigB^5^ | -5,0 | Translation initiation factor IF-3 | Translation |
| *lmo1328* | *truB* |  | -5,0 | tRNA pseudouridine synthase B | Translation |
| *lmo2429* | *fhuC* | Fur^2^, SigB^5^ | -5,0 | Ferrichrome transport ATP-binding protein FhuC | Inorganic ion transport and metabolism; Coenzyme transport and metabolism |
| *lmo0807* | *potA* |  | -5,0 | Putrescine transport ATP-binding protein PotA | Amino acid transport and metabolism |
| *lmo0704* |  |  | -4,9 | Hypothetical protein | Not in COGs |
| *lmo1784* | *rpmI* |  | -4,9 | 50S ribosomal protein L35 | Translation |
| *lmo0427*^¥^ |  |  | -4,9 | PTS system, fructose-specific IIB component | Carbohydrate transport and metabolism |
| *lmo0864* |  |  | -4,9 | Predicted glycoside hydrolase | Carbohydrate transport and metabolism |
| *lmo0543* |  |  | -4,8 | PTS system, glucitol/sorbitol-specific IIB component and second of two IIC components | Carbohydrate transport and metabolism |
| *lmo1417* |  |  | -4,8 | Membrane protein, putative | General function prediction only |
| *lmo0873* |  |  | -4,8 | Transcriptional antiterminator, PTS system IIA 2 domain protein | Transcription;  Carbohydrate transport and metabolism; Signal transduction mechanisms |
| *lmo2655* | *rpsG* |  | -4,8 | 30S ribosomal protein S7 | Translation |
| *lmo0692* | *cheA* |  | -4,8 | Two-component sensor histidine kinase CheA | Cell motility;  Signal transduction mechanisms |
| *lmo0357* |  |  | -4,8 | PTS system, fructose-specific IIA component | Carbohydrate transport and metabolism; Signal transduction mechanisms |
| *lmo2154* | *nrdF* |  | -4,8 | Ribonucleotide-diphosphate reductase subunit beta | Nucleotide transport and metabolism |
| *lmo1765* | *purH* |  | -4,8 | Bifunctional phosphoribosylaminoimidazolecarboxamide formyltransferase/IMP cyclohydrolase | Nucleotide transport and metabolism |
| *lmo2848* |  |  | -4,7 | L-rhamnose isomerase | Carbohydrate transport and metabolism |
| *lmo2797*^¥§^ |  |  | -4,7 | PTS system, mannitol-specific IIA component | Carbohydrate transport and metabolism; Signal transduction mechanisms |
| *lmo0485*^§^ |  |  | -4,7 | Putative nitroreductase family protein | Energy production and conversion |
| *lmo0033* |  |  | -4,7 | Glycosyl hydrolase, family 9 | Not in COGs |
| *lmo2633* | *rpsJ* |  | -4,7 | 30S ribosomal protein S10 | Translation |
| *lmo0688* |  |  | -4,6 | Glycosyl transferase, group 2 family protein | Cell wall/membrane biogenesis;  General function prediction only |
| *lmo0718*^§^ |  |  | -4,5 | Hypothetical protein | Not in COGs |
| *lmo2667*^¥^ |  |  | -4,5 | PTS system, galactitol-specific IIA component | Carbohydrate transport and metabolism; Signal transduction mechanisms |
| *lmo2153* |  |  | -4,5 | Flavodoxin | Energy production and conversion |
| *lmo1654* |  |  | -4,5 | Putative cellsurface protein | Not in COGs |
| *lmo0689* | *cheV* | CodY^8^ | -4,4 | Chemotaxis protein CheV | Cell motility;  Signal transduction mechanisms |
| *lmo2798*^¥§^ |  |  | -4,4 | Hydrolase, haloacid dehalogenase-like family | General function prediction only |
| *lmo0124* |  |  | -4,4 | Hypothetical protein | Not in COGs |
| *lmo1031* |  |  | -4,4 | Unknown pentose isomerase | Carbohydrate transport and metabolism |
| *lmo1254*^¥§^ |  |  | -4,3 | Trehalose-6-phosphate hydrolase | Carbohydrate transport and metabolism |
| *lmo1262* |  |  | -4,3 | Ans operon transcriptional repressor | Transcription |
| *lmo2348* | *tcyL* |  | -4,3 | L-Cystine ABC transporter, permease protein TcyL | Amino acid transport and metabolism |
| *lmo2666*^¥^ |  | CodY^8^ | -4,3 | PTS system, galactitol-specific IIB component | Carbohydrate transport and metabolism |
| *lmo0499* |  |  | -4,2 | Ribulose-phosphate 3-epimerase | Carbohydrate transport and metabolism |
| *lmo1728* |  |  | -4,2 | Sugar phosphorylase | Carbohydrate transport and metabolism |
| *lmo2138* |  |  | -4,2 | Transcriptional antiterminator of lichenan operon, BglG family | Transcription |
| *lmo1336* |  |  | -4,2 | 5-formyltetrahydrofolate cyclo-ligase | Coenzyme transport and metabolism |
| *lmo1142* |  |  | -4,2 | Hypothetical protein | Energy production and conversion |
| *lmo0604* |  | VirRS^10^ | -4,2 | Hypothetical protein | Not in COGs |
| *lmo0383* |  |  | -4,1 | Methylmalonate-semialdehyde dehydrogenase | Energy production and conversion |
| *lmo2354* |  |  | -4,1 | Exoenzymes regulatory protein AepA precursor | General function prediction only |
| *lmo2261*^§^ |  | CtsR^7^ | -4,1 | Hypothetical protein | General function prediction only |
| *lmo0856* | *murF* |  | -4,1 | UDP-N-acetylmuramoylalanyl-D-glutamyl-2,6-diamino pimelate-D-alanyl-D-alanyl ligase | Cell wall/membrane biogenesis |
| *lmo2656* | *rpsL* |  | -4,1 | 30S ribosomal protein S12 | Translation |
| *lmo2349* | *tcyK* |  | -4,0 | L-Cystine ABC transporter, periplasmic cystine-binding protein TcyK | Amino acid transport and metabolism; Signal transduction mechanisms |
| *lmo2766* |  |  | -4,0 | Phosphosugar-binding transcriptional regulator, RpiR family | Transcription |
| *lmo1194* | *cbiD* |  | -4,0 | Cobalt-precorrin-6A synthase | Coenzyme transport and metabolism |
| *lmo1453* |  |  | -4,0 | Hypothetical protein | General function prediction only |
| *lmo1497* | *udk* |  | -4,0 | Uridine kinase | Nucleotide transport and metabolism |
| *lmo2847* |  |  | -4,0 | Rhamnulose-1-phosphate aldolase | Carbohydrate transport and metabolism |

a Hemin stressed vs. non-stressed *L. monocytogenes* EGD-e cells

^b^ Information from Listeriomics website (listeriomics.pasteur.fr)

^1^ (Torres et al., 2007)

^2^ (McLaughlin et al., 2012)

^3^ (Rea et al., 2005)

^4^ (van der Veen et al., 2010)

^5^ (Hain et al., 2008)

^6^ (Nielsen et al., 2012)

^7^ (Hu et al., 2007)

^8^ (Bennett et al., 2007)

^9^ (Milohanic et al., 2003)

^10^ (Mandin et al., 2005)

^¤^ Genes coding for hypothetical proteins identified as up-regulated in blood by (Toledo-Arana et al., 2009)

^¥^ Genes found to be as well up- or down- regulated during anaerobic growth by (Muller-Herbst et al., 2014)

^§^ Genes found to be as well up- or down-regulated during growth under low oxygen by (Toledo-Arana et al., 2009)

**References:**

Bennett, H.J., Pearce, D.M., Glenn, S., Taylor, C.M., Kuhn, M., Sonenshein, A.L., et al. (2007). Characterization of relA and codY mutants of Listeria monocytogenes: identification of the CodY regulon and its role in virulence. *Mol Microbiol* 63(5)**,** 1453-1467. doi: 10.1111/j.1365-2958.2007.05597.x.

Hain, T., Hossain, H., Chatterjee, S.S., Machata, S., Volk, U., Wagner, S., et al. (2008). Temporal transcriptomic analysis of the Listeria monocytogenes EGD-e sigmaB regulon. *BMC Microbiol* 8**,** 20. doi: 10.1186/1471-2180-8-20.

Hu, Y., Raengpradub, S., Schwab, U., Loss, C., Orsi, R.H., Wiedmann, M., et al. (2007). Phenotypic and transcriptomic analyses demonstrate interactions between the transcriptional regulators CtsR and Sigma B in Listeria monocytogenes. *Appl Environ Microbiol* 73(24)**,** 7967-7980. doi: 10.1128/AEM.01085-07.

Mandin, P., Fsihi, H., Dussurget, O., Vergassola, M., Milohanic, E., Toledo-Arana, A., et al. (2005). VirR, a response regulator critical for Listeria monocytogenes virulence. *Mol Microbiol* 57(5)**,** 1367-1380. doi: 10.1111/j.1365-2958.2005.04776.x.

McLaughlin, H.P., Xiao, Q., Rea, R.B., Pi, H., Casey, P.G., Darby, T., et al. (2012). A putative P-type ATPase required for virulence and resistance to haem toxicity in Listeria monocytogenes. *PLoS One* 7(2)**,** e30928. doi: 10.1371/journal.pone.0030928.

Milohanic, E., Glaser, P., Coppee, J.Y., Frangeul, L., Vega, Y., Vazquez-Boland, J.A., et al. (2003). Transcriptome analysis of Listeria monocytogenes identifies three groups of genes differently regulated by PrfA. *Mol Microbiol* 47(6)**,** 1613-1625.

Muller-Herbst, S., Wustner, S., Muhlig, A., Eder, D., T, M.F., Held, C., et al. (2014). Identification of genes essential for anaerobic growth of Listeria monocytogenes. *Microbiology* 160(Pt 4)**,** 752-765. doi: 10.1099/mic.0.075242-0.

Nielsen, P.K., Andersen, A.Z., Mols, M., van der Veen, S., Abee, T., and Kallipolitis, B.H. (2012). Genome-wide transcriptional profiling of the cell envelope stress response and the role of LisRK and CesRK in Listeria monocytogenes. *Microbiology* 158(Pt 4)**,** 963-974. doi: 10.1099/mic.0.055467-0.

Rea, R., Hill, C., and Gahan, C.G. (2005). Listeria monocytogenes PerR mutants display a small-colony phenotype, increased sensitivity to hydrogen peroxide, and significantly reduced murine virulence. *Appl Environ Microbiol* 71(12)**,** 8314-8322. doi: 10.1128/AEM.71.12.8314-8322.2005.

Toledo-Arana, A., Dussurget, O., Nikitas, G., Sesto, N., Guet-Revillet, H., Balestrino, D., et al. (2009). The Listeria transcriptional landscape from saprophytism to virulence. *Nature* 459(7249)**,** 950-956. doi: 10.1038/nature08080.

Torres, V.J., Stauff, D.L., Pishchany, G., Bezbradica, J.S., Gordy, L.E., Iturregui, J., et al. (2007). A Staphylococcus aureus regulatory system that responds to host heme and modulates virulence. *Cell Host Microbe* 1(2)**,** 109-119. doi: 10.1016/j.chom.2007.03.001.

van der Veen, S., van Schalkwijk, S., Molenaar, D., de Vos, W.M., Abee, T., and Wells-Bennik, M.H. (2010). The SOS response of Listeria monocytogenes is involved in stress resistance and mutagenesis. *Microbiology* 156(Pt 2)**,** 374-384. doi: 10.1099/mic.0.035196-0.

1. [↑](#endnote-ref-1)
